# A New Mouse Model Related to SCA14 Carrying a Pseudosubstrate Domain Mutation in PKCγ Shows Perturbed Purkinje Cell Maturation and Ataxic Motor Behavior

**DOI:** 10.1523/JNEUROSCI.1946-20.2021

**Published:** 2021-03-03

**Authors:** Etsuko Shimobayashi, Josef P. Kapfhammer

**Affiliations:** Anatomical Institute, Department of Biomedicine, University of Basel, Basel, CH-4056, Switzerland

**Keywords:** ataxic motor behavior, cerebellar Purkinje cell, dendritic development, neurodegenerative diseases, protein kinase C gamma, spinocerebellar ataxia

## Abstract

Spinocerebellar ataxias (SCAs) are diseases characterized by cerebellar atrophy and loss of Purkinje neurons caused by mutations in diverse genes. In SCA14, the disease is caused by point mutations or small deletions in protein kinase C γ (PKCγ), a crucial signaling protein in Purkinje cells. It is still unclear whether increased or decreased PKCγ activity may be involved in the SCA14 pathogenesis. In this study, we present a new knock-in mouse model related to SCA14 with a point mutation in the pseudosubstrate domain, PKCγ-A24E, known to induce a constitutive PKCγ activation. In this protein conformation, the kinase domain of PKCγ is activated, but at the same time the protein is subject to dephosphorylation and protein degradation. As a result, we find a dramatic reduction of PKCγ protein expression in *PKC*γ*-A24E* mice of either sex. Despite this reduction, there is clear evidence for an increased PKC activity in Purkinje cells from *PKC*γ*-A24E* mice. Purkinje cells derived from PKCγ-A24E have short thickened dendrites typical for PKC activation. These mice also develop a marked ataxia and signs of Purkinje cell dysfunction making them an interesting new mouse model related to SCA. Recently, a similar mutation in a human patient was discovered and found to be associated with overt SCA14. RNA profiling of *PKC*γ*-A24E* mice showed a dysregulation of related signaling pathways, such as mGluR1 or mTOR. Our results show that the induction of PKCγ activation in Purkinje cells results in the SCA-like phenotype indicating PKC activation as one pathogenetic avenue leading to a SCA.

**SIGNIFICANCE STATEMENT** Spinocerebellar ataxias (SCAs) are hereditary diseases affecting cerebellar Purkinje cells and are a one of neurodegenerative diseases. While mutation in several genes have been identified as causing SCAs, it is unclear how these mutations cause the disease phenotype. Mutations in PKCγ cause one subtype of SCAs, SCA14. In this study, we have generated a knock-in mouse with a mutation in the pseudosubstrate domain of PKCγ, which keeps PKCγ in the constitutive active open conformation. We show that this mutation leading to a constant activation of PKCγ results in a SCA-like phenotype in these mice. Our findings establish the constant activation of PKC signaling as one pathogenetic avenue leading to an SCA phenotype and a mechanism causing a neurodegenerative disease.

## Introduction

Spinocerebellar ataxia 14 (SCA14; OMIM 605361) is a rare autosomal dominant neurodegenerative disease caused by protein kinase Cγ gene (*PRKCG*) mutations, the incidence of which is 1%–4% of all autosomal dominant cerebellar ataxias (Verbeek et al., 2005; Chen et al., 2012).

PKCγ is a serine/threonine kinase dominantly expressed in cerebellar Purkinje cells and playing an important role for Purkinje cell functions (Chopra et al., 2018; Hirai, 2018). Increased PKC activity has a strong negative impact on Purkinje cell dendritic outgrowth and development in organotypic slice cultures (Metzger and Kapfhammer, 2000), whereas Purkinje cells from PKCγ-deficient mice show no gross morphologic abnormalities (Chen et al., 2005). SCA14 is a dominantly inherited disease; therefore, gain of function or a dominant negative function rather than a loss of function of PKCγ might cause SCA14.

To date, >40 missense mutations or deletions in *PRKCG* have been reported, and many mutations have been found in the cysteine-rich regulatory domain (C1A and C1B domain) while some other mutations have been found in the pseudosubstrate domain, the calcium binding C2 domain or the kinase domain (Adachi et al., 2008). The question is how all these mutations in *PRKCG* are linked to the disease phenotype. For some mutations, an increased PKCγ kinase activity was shown pointing toward a gain of function phenotype (Verbeek et al., 2005; Adachi et al., 2008). In contrast, other SCA14 mutations, especially in the C1 domain, are functionally defective because of decreased binding to diacylglycerol pointing toward a loss of function phenotype (Verbeek et al., 2008). These findings suggest that pathology in SCA14 is not simply because of one single mechanism but rather the result of complex mechanisms involving dysregulation of PKCγ (Shimobayashi and Kapfhammer, 2018; Wong et al., 2018).

We have previously created a transgenic mouse model expressing a kinase domain mutant PKCγ with a constitutive activation. In this mouse model, we found subtle changes of the Purkinje cell dendritic tree and a mild ataxia (Ji et al., 2014; Trzesniewski et al., 2019). In these mice, there are still two normal alleles of PKCγ present, making it difficult to compare the model to human disease. We have now created a new knock-in mouse model with a mutation in the pseudosubstrate domain. This autoinhibitory domain is crucial for regulating PKCγ activity by preventing access of substrates to the kinase domain (Newton, 2018). The pseudosubstrate domain is only dissociated from the kinase domain after binding of diacylglycerol (Baffi et al., 2019), allowing PKCγ substrates to access the kinase domain and become phosphorylated (Newton, 2018). This “open-active” conformation of PKCγ is then subject to dephosphorylation and degradation. This activation cycle of PKCγ is well controlled in Purkinje cells and critical for the regulation of dendritic development, synaptic function in LTD, and synapse formation (Kano et al., 1995; Saito and Shirai, 2002). Mutations in the autoinhibitory pseudosubstrate domain reduce its affinity to the kinase domain and will increase kinase activity but also induce PKCγ dephosphorylation and degradation (Pears et al., 1990).

In this study, we introduced a mutation in the pseudosubstrate sequence, which keeps PKCγ in the constitutive active open conformation; and we generated a knock-in mouse carrying this mutation. Another pseudosubstrate domain mutation at the same A24 position (A24T) was recently identified in a human SCA14 patient (Chelban et al., 2018). We found that the A24E mutation indeed induced increased PKC activity but at the same time made PKCγ very prone to degradation. Purkinje cells expressing the mutated PKCγ showed compromised dendritic development; and in the corresponding knock-in mouse model, we observed a marked ataxia, altered Purkinje cell morphology, and abnormal climbing fiber (CF) termination. Gene expression profiling revealed alterations in related signaling pathways, such as Type 1 metabotropic glutamate receptor (mGluR1) or mTOR. Our results support the concept that the regulation of PKC activity is crucial for Purkinje cell function and one important contributor to the pathogenesis of SCA14 and other SCAs.

## Materials and Methods

### 

#### Plasmid construction

Mutated *PRKCG* genes were generated as described previously (Shimobayashi et al., 2016), using the following mutagenic primers: A24E forward primer, 5′-TTTGCAGAAAGGGGGAGCTGAGGCAGAAGGTGGT-3′; A24E reverse primer, 5′-ACCACCTTCTGCCTCAGCTCCCCCTTTCTGCAAA-3′; A24T forward primer, 5′-TTTGCAGAAAGGGGACTCTGAGGCAGAAGGTGGT-3′; and A24T reverse primer, 5′-ACCACCTTCTGCCTCAGAGTCCCCTTTCTGCAAA-3′.

The PCR products sequence were confirmed by DNA sequencing (Microsynth).

#### PKCγ-A24E overexpression in HeLa cells and HEK293T cells

Human *PRKCG* gene was obtained from Origene in pCMV6-XL4 (pCMV6-XL4-*PRKCG*); 5 µg of pCMV-GFP control, pCMV-PKCγ-Wt, or pCMV-PKCγ-A24E was transfected into HeLa cells (ATCC, RRID: CVCL_0030) or HEK293T cells (ATCC, RRID: CVCL_0063) using X-fect Transfection Reagent (Takara). After 24 or 48 h, cells were fixed with 4% PFA and stained with following antibodies: mouse anti-GFP (1:1000, Abcam; ab290), rabbit anti-PKCγ (1:1000, Santa Cruz Biotechnology; sc-211), and DAPI. The staining was visualized with AlexaFluor-568 goat anti-rabbit (1:500, Invitrogen; A11011) and AlexaFluor-488 goat anti-mouse (1:500, Invitrogen; A11001). Images were acquired with confocal microscopy (Carl Zeiss, LSM700) equipped with solid-state lasers using a Plan-Apochromat 100×/1.3 Oil DIC M27 objective (Carl Zeiss). To monitor protein half-life by cycloheximide chase, HEK293T cells were treated with 35 µg/ml cycloheximide (Sigma Millipore) in DMSO at 48 h after transfection, cells were collected at multiple time points (0 min, 30 min, 90 min, 240 min, and 24 h) after treatment. To inhibit protein degradation via ubiquitin proteasome pathway, 5 μm MG132 (Sigma Millipore) was added to cells at 48 h after transfection, and samples were collected at 24 h after MG132 treatment. Each sample was homogenized on ice using an ultrasound probe in ice-cold RIPA buffer (50 mm Tris-HCl, pH 7.4, 0.15 M NaCl, 0.25% deoxycholic acid/sodium deoxycholate, 1% NP-40, 1 mm EDTA) added protease- and phosphatase-inhibitors (Roche Diagnostics) and then centrifuged at 7500 × *g* for 15 min. Protein concentration was determined using the BCA kit (Bio-Rad), and 50 µg of each sample was subjected to SDS-PAGE for analyzing protein expression.

#### Generation of transgenic mice

Animal experiments were conducted in accordance with the EU Directive 2010/63/EU for animal experiments and were reviewed and permitted by Swiss authorities. All experiments were done for both male and female mice. The point mutations (c. 71C > A; p. Ala 24 Glu, c. 78G > A; p. Arg 26 Arg) in the *PRKCG* gene (chromosome 7, 1.93 cM) were introduced into FVB background mice. Protospacer Adjacent Motif (PAM) sequence, 78G > A mutation that does not change amino acid was introduced to prevent the donor DNA from being a suitable target for Cas9 cleavage. The knock-in mice using Cas9/CRISPR engineering system were generated at the Center of Transgenic Models, University of Basel, with the rapid oocyte injection method. Alt-R CRISPR-Cas9 crRNA was designed specific to exon 1 of *PRKCG* (T TGC AGA AAG GGG GCG CTG) upstream of PAM sequence; Alt-R CRISPR-Cas9 tracrRNA and donor DNA (CAC CAG ATG AAG TCG GTA CAG TGA CTG CAG AAG GTT GGC TGC TTG AAG AAA CGA GCG GTG AAC TTG TGG CTC TTC ACC TCG TGG ACC ACC TTC TGT CTC AGC TCC CCC TTT CTG CAA AAC AGG GGT CGG GGT CCC CCC TCT GAG TCG CCT CCG CCA GGG CCC AGA CCC GCC ATG) was obtained from Integrated DNA Technologies. Crispr RNA, Cas9, and donor DNA were injected into FVB zygotes at the pronuclear site, and surviving embryos were transferred into pseudo-pregnant mothers. To identify founders, genotyping with genomic DNA samples from biopsies was performed by PCR. The primers for genotyping were as follows: forward primer, 5′ TCC TTC CTA TCT CAG AGT CTG CG 3′; and reverse primer, 5′ GTT CCC AAG TCC CCT CCT TTT CC 3′ (Microsynth). Then, the mutations at 71C > A and 78G > A in the *PRKCG* were confirmed by DNA sequencing. The fragment for sequencing was obtained by PCR with genomic DNA samples and primers as mentioned above. The confirmed point mutation knock-in founders were crossed with FVB (Janvier labs) mice to obtain Wt, heterozygous (Het), and homozygous (Homo) *PKC*γ*-A24E* mice.

#### Real-time qPCR

RNA was purified from the cerebellum of control and *PKC*γ*-A24E* mice at different ages using the RNeasy Mini-kit (QIAGEN) following the instructions of the manufacturer; 1 µg of total RNA was used for reverse transcription reaction with SuperScript IV Reverse Transcriptase (Takara). Real-time qPCR was performed on a StepOne real-time PCR system (Applied Biosystems) using the SYBR Green master mix (Applied Biosystems). The following primers and reaction conditions were used: mouse *PRKCG* forward primer, 5′-CAAAACAGAAGACAAAGACC-3′; mouse *PRKCG* reverse primer, 5′-GGCCTTGAGTAGCTCTGAGACA-3′; *GAPDH* forward primer, 5′-AACTTTGGCATTGTGGAAGG-3′; and *GAPDH* reverse primer, 5′-ACACATTGGGGGTAGGAACA-3′.

Reaction conditions are as follows: 1 cycle of (10 min at 95°C), 40 cycles of (15 s at 95°C and 60 s at 65°C), and 1 cycle of (15 s at 95°C, 30 s at 72°C and 15 s at 95°C).

Reactions were quantified by the relative standard curve system and the cycle threshold method using the SDS2.2 software (Applied Biosystems). A relative quantitation value for each sample from the triplicates of that sample was calculated for each gene. The data were analyzed with GraphPad Prism software.

#### Western blot analysis

Wt and *PKC*γ*-A24E* mice at different ages were killed by an overdose of pentobarbital; then the cerebellum was quickly dissected and frozen in liquid nitrogen. Samples were homogenized as mentioned above. Organotypic slice culture samples were harvested and transferred to RIPA buffer-added protease and phosphatase inhibitors (Roche Diagnostics); 25 nm okadaic acid (Tocris Bioscience; 1136) was added for protein phosphatase 1 and 2A inhibition. Protein concentrations were determined using the BCA kit (Bio-Rad), and 50 µg of each sample was subjected to SDS-PAGE. The separated proteins were transferred to a nitrocellulose membrane using a semidry blotting machine (Bio-Rad). After blotting, membranes were blocked with 5% BSA (Sigma Millipore) in TBS for 1 h and incubated with appropriate primary antibodies, rabbit anti-PKCγ (1:1000, Santa Cruz Biotechnology; sc-211), rabbit anti-PKCα (1:1000, Invitrogen; PA5-17551), rabbit anti-phospho-(Ser) PKC substrate (1:1000, Cell Signaling Technology; #2261), rabbit anti-ubiquitin (1:1000, Cell Signaling Technology; #43124), rabbit anti-NMDA receptor 1 (GluN1; 1:500, Cell Signaling Technology; #5704), rabbit anti-myristoylated alanine-rich C kinase substrate (MARCKS) (1:1000, Invitrogen; PA 5-105296), rabbit anti-phospho-NMDA receptor 1 (GluN1-Ser890; 1:1000, Cell Signaling Technology; #3381), rabbit anti-phospho-(Ser152, Ser156)-MARCKS (1:1000, Invitrogen; PA1-4629), rabbit anti-Homer3 (1:1000, Invitrogen; PA5-59383), rabbit anti-Rmdn3 (1:1000, ABclonal; A5820) or mouse anti-Actinβ (1:2000, Sigma Millipore; A5441). After washing with TBS plus 0.5% Triton X-100 (TBS-T), membranes were incubated with secondary antibodies. Secondary antibodies were as follows: IRDye 800CW Donkey anti-mouse (1:10 000, LI-COR Biosciences; 926-32 212) and IRDye 680LT Donkey anti-rabbit (1:10,000, LI-COR Biosciences; 926-68 023). After washing, signal was detected using a LI-COR Odyssey instrument and software (LI-COR Biosciences).

#### Organotypic slice cultures

Slice cultures were prepared as described previously (Gugger et al., 2012). Briefly, mice were decapitated at postnatal day 8 (P8), their brains were aseptically removed, and the cerebellum was dissected in ice-cold preparation medium: MEM, 1% Glutamax (Invitrogen), pH 7.3. Sagittal sections (350 µm thickness) were cut on a McIllwain tissue chopper under aseptic conditions. Slices were separated, transferred onto permeable membranes (Millicell-CM, Millipore), and incubated on a layer of neurobasal medium: Neurobasal A medium (Invitrogen) supplemented with B27 supplement (Invitrogen) and Glutamax (Invitrogen), pH 7.3, in a humidified atmosphere with 5% CO_2_ at 37°C. The medium was changed every 2-3 d; 300 nm PMA (Tocris Bioscience) was added for PKC activation 24 h before slices were fixed; 10 μm Gö6983 (Tocris Bioscience) was added to the medium at each medium change for PKC inhibition, starting at 3 DIV (DIV3). Slices were kept in culture for a total of 7 d and analyzed with Western blot and immunohistochemical staining. To monitor protein degradation, slices were treated with 50 µg/ml cycloheximide (Sigma Millipore) or 10 μm MG132 (Sigma Millipore) or both at DIV7, and protein was extracted at DIV8.

#### Histology and immunohistochemistry

Immunohistochemistry was performed as described previously (Shimobayashi et al., 2016). For the analysis of the cerebellar sections, Wt and *PKC*γ*-A24E* mice were killed by perfusion with 4% PFA; then the cerebellum was removed, fixed with 4% PFA for 1 d at 4°C, and cryoprotected in 30% sucrose for 1 d at 4°C. Cerebella were frozen in isopentane on dry ice and embedded in optimal cutting temperature compound, and parasagittal cryosections of 20 µm thickness were cut on a Leica Microsystems CM1900 cryostat and used for immunohistochemistry. Organotypic slice cultures were fixed at DIV7 in 4% PFA overnight at 4°C. All reagents were diluted in 100 mm PB, pH 7.3. Cryosections or organotypic slices were incubated in blocking solution (0.5% Triton X-100, 3% normal goat serum [Invitrogen]) for 1 h to permeabilize the tissue and block nonspecific antigen binding. Primary antibodies were added in blocking solution and incubated overnight at 4°C with the following antibodies: mouse anti-calbindin D28K (1:1000, Swant; #300), guinea pig anti-vesicular glutamate transporter 2 (vGlut2) (1:1000, Millipore; AB2251-I), rabbit anti-PKCγ (1:1000, Santa Cruz Biotechnology; sc-211). The staining was visualized with AlexaFluor-568 goat anti-rabbit (1:500, Invitrogen; A11011) and AlexaFluor-488 goat anti-mouse (1:500, Invitrogen; A11001). Stained slices or sections were mounted on glass slides using Mowiol. Cryosections or organotypic slices were viewed on an Olympus AX-70 microscope equipped with a Spot digital camera. Recorded images were adjusted for brightness and contrast with Photoshop image processing software.

#### Dissociated cerebellar cultures

Dissociated cerebellar cultures were prepared from mice essentially as described previously (Shimobayashi and Kapfhammer, 2018). After starting the culture, half of medium were changed twice a week. For PKC activation or inhibition assay, 15 nm PMA (Tocris Bioscience) for PKC activation or 5 μm Gö6983 (Tocris Bioscience) for PKC inhibition was added to the medium at each change starting at DIV7 or DIV4. For plasmid transfection, L7-based expression vectors were constructed using the primers mentioned above.

Transfections were performed as described previously (Shimobayashi et al., 2016) using the 4D-Nucleofector System (Lonza Walkersville) according to the manufacturer's instructions.

#### Immunohistochemistry of dissociated cerebellar cells

After 14-18 d, cells were fixed in 4% PFA for 1 h at 4°C. All reagents were diluted in 100 mm PB, pH 7.3. Cells were incubated in blocking solution (0.5% Triton X-100, 3% normal goat serum [Invitrogen]) for 30 min at room temperature. Two different primary antibodies were simultaneously added to the cells in fresh blocking solution and incubated for 30 min at room temperature. After washing in PB, secondary antibodies were added to the slices in PB containing 0.1% Triton X-100 for 30 min at room temperature. For the analysis of vector expression in Purkinje cells, mouse anti-Calbindin D-28K (1:1000, Swant; #300) and polyclonal rabbit anti-GFP (1:1000, Abcam; ab6556) were used as primary antibodies, and AlexaFluor-568 goat anti-rabbit (1:1000, Invitrogen; A11011) and AlexaFluor-488 goat anti-mouse (1:1000, Invitrogen; A11001) were used as secondary antibodies to visualize Purkinje cells (Ji et al., 2014). Stained cells were viewed on an Olympus AX-70 microscope equipped with a Spot digital camera or were acquired with confocal microscopy (Carl Zeiss, LSM710) equipped with solid state lasers using a LD Plan-Neofluar 40× objective (Carl Zeiss). Recorded images were adjusted for brightness and contrast with Photoshop image processing software.

#### Golgi-Cox staining

Mice from P14 to 1 year old were used for the Golgi-Cox study. The FD Rapid GolgiStain Kit (FD Neuro Technologies) was used for Golgi staining. Mice were killed and perfused with 4% PFA. The cerebellum was collected in 4 ml of impregnation Solutions A and B according to the instructions. After 3 weeks of impregnation, the cerebellum was placed in Solution C from the FD Rapid GolgiStain kit and stored at room temperature for 3 d. Then, cerebella were frozen in isopentane on dry ice and kept in −80°C until use. Sections were cut at 100 μm using a Leica Microsystems CM1900 cryostat, collected and mounted on gelatin-coated slides using Solution C, and dried at room temperature overnight in the dark place. The next day, the staining was developed using Solutions D and E and distilled water at a 1:1:2 concentrations. The sections were then dehydrated in increasing alcohol concentrations and coverslipped using Eukitt mounting medium (Sigma Millipore). Slides were viewed on bright field microscope and images were taken using a Spot digital camera.

#### Experimental design and statistical analysis

The age of the mice for each experiment is shown in Table 1. Statistical comparisons were made using the GraphPad Prism 8.3.1 software package (GraphPad Software). The quantification of Purkinje cell dendritic tree size was done as previously described (Gugger et al., 2012). Purkinje cells that had a dendritic tree isolated from its surroundings were selected for analysis. Cells were photographed with a digital camera. An image analysis program (ImageJ, https://imagej.nih.gov/ij/) was used to trace the outline of the Purkinje cell dendritic trees yielding the area covered by the dendritic tree. More than 20 cells were acquired from at least three independent experiments were analyzed. The statistical significance was assessed by nonparametric Mann–Whitney's test. CIs were 95%, statistical significance when *p* < 0.05. Graphical data are represented as the mean ± SEM.

**Table 1.** Summary of the ages of mice used for each experiment

Figure	Age of mice
3*E*	4- and 6-week-old
3*F*	7-week-old
6*A*	40-week-old
6*B*	40-week-old
6*C*	4- and 53-week-old
6*D*	4- and 40-week-old
7, Beam walk	3- and 6-month-old
7, Rotarod	3- and 6-month-old
7, Footprint	9-month-old
8	5- and 7-week-old

**Table 2.** Analysis of the phosphoprotein localization*^a^*

Location	Total phosphoprotein	Upregulated or downregulated in A24E	Upregulated in A24E (*p* < 0.05)	Upregulated phosphoprotein out of total phosphoprotein (%)
Cytoplasm	1200	73	44	3.667
Plasma membrane	532	44	33	6.203
Nucleus	795	46	24	3.019
Extracellular space	85	6	2	2.353
Other	193	5	2	1.036
Total	2805	174	109	3.886

^a^Cellular localizations of proteins with significantly increased phosphorylation in homo *PKC*γ*-A24E* mice.

To quantify the protein expression in Western blots, the immunoreactivity of each sample was normalized to the actinβ signal, and the ratio was evaluated and then normalized to Wt control using LI-COR software (LI-COR Biosciences). Protein or phospho-protein expression differences between each sample were analyzed using the two-tailed Mann–Whitney test.

Statistical significance of the Footprint pattern test, the Rotarod test, and the Walking beam test was analyzed using ANOVA models, and Wt and *PKC*γ*-A24E* mice groups were analyzed by two-way ANOVA test with Bonferroni correction.

##### Behavioral testing

##### Rotarod

To measure motor function, mice were placed on an accelerating rotarod (Rotamex-5, Columbus Instruments), and the speed of rotation was increased from 2 to 52 revolutions per minute over 4 min. The latency to fall from the rotarod was recorded. Data were collected for 5 trials per day after a training period of 5 trials per day for 4-5 d. The two-way ANOVA test with Bonferroni correction was conducted to examine the main effect of genotype on each day.

##### Walking beam test

In this test, the mice had to traverse an 80-cm-long and 8-mm-wide wooden bar. Every slip of a hind paw was recorded and counted. After a 1 d training period, data were collected for 10 trials on 4 or 5 different days. The statistical significance was assessed by two-way ANOVA test with Bonferroni correction.

##### Footprint pattern test

The footprint patterns were evaluated at 9-month-old Wt and *PKC*γ*-A24E* mice groups. Mouse paws were painted with nontoxic ink, and mice were placed at one end of a dark tunnel. Mice walked through the tunnel, and their footprints were analyzed for the width and length of each step. The statistical significance was assessed by two-way ANOVA test with Bonferroni correction.

All data were analyzed using GraphPad Prism software. Statistical significance was assumed as follows: *p < 0.05, **p < 0.01, ***p < 0.001, or ****p < 0.0001.

#### Total RNA extraction and total RNA sequence

The cerebellum was isolated, and quickly slices of 500 μm thickness were made. Purkinje layer and molecular layer were microdissected and harvested in RNAlater. Cerebellum lysates from 3 mice in each group were collected for total RNA isolation, following the instructions of the manufacturers of the Trizol and RNeasy Lipid Tissue Mini Kit (QIAGEN). Samples were eluted in 15 μl of RNase-free H_2_O, quantified using a Nanodrop ND-1000 (Thermo Fisher Scientific) spectrophotometer. RNA qualities were checked using Agarose Gel Electrophoresis and Bioanalyzer 2100 (Agilent Technologies). All samples were adjusted as 100 ng/μl and only used when RNA integrity number was ≥ 7.1. Library preparation and sequencing were performed at the Quantitative Genomics Facility of the University of Basel and the ETH Zurich at Basel.

#### Phosphoproteomics

The cerebella were isolated and quickly frozen with liquid nitrogen. Protein was extracted with lysis buffer, including PhosSTOP (Roche Diagnostics); then protein concentration was measured with the BCA Protein Assay kit (Invitrogen); 1 mg of mouse tissue was lysed in 80 μl of 8 m urea, 0.1 m ammonium bicarbonate, phosphatase inhibitors (Sigma Millipore; P5726 and P0044) by sonication (Bioruptor, 10 cycles, 30 s on/off, Diagenode), and proteins were digested as described previously (PMID:27345528). Pewptide samples were enriched for phosphorylated peptides using Fe(III)-IMAC cartridges on an AssayMAP Bravo platform as recently described (PMID: 28107008). Phospho-enriched peptides were resuspended in 0.1% aqueous formic acid and subjected to LC-MS/MS analysis using an Orbitrap Fusion Lumos Mass Spectrometer fitted with an EASY-nLC 1200 (Thermo Fisher Scientific). LC-MS was performed by Alexander Schmidt in the Biocenter of the University of Basel, Switzerland.

#### Ingenuity pathway analysis (IPA)

IPA is a web-based biological analysis tool for omics data, and genomic data. We applied RNA sequence data and phosphoproteomics data into QIAGEN's IPA software (https://www.qiagenbioinformatics.com/products/ingenuity-pathway-analysis/) and performed a core analysis that indicates not only direct but also indirect relationships between genes and proteins. The IPA software shows the overlapping canonical pathways, upstream regulators, and affected genes/proteins networks.

## Results

### A24E mutant PKCγ is unstable, aggregates, and is partially degraded by proteasomes

**Figure 1.**
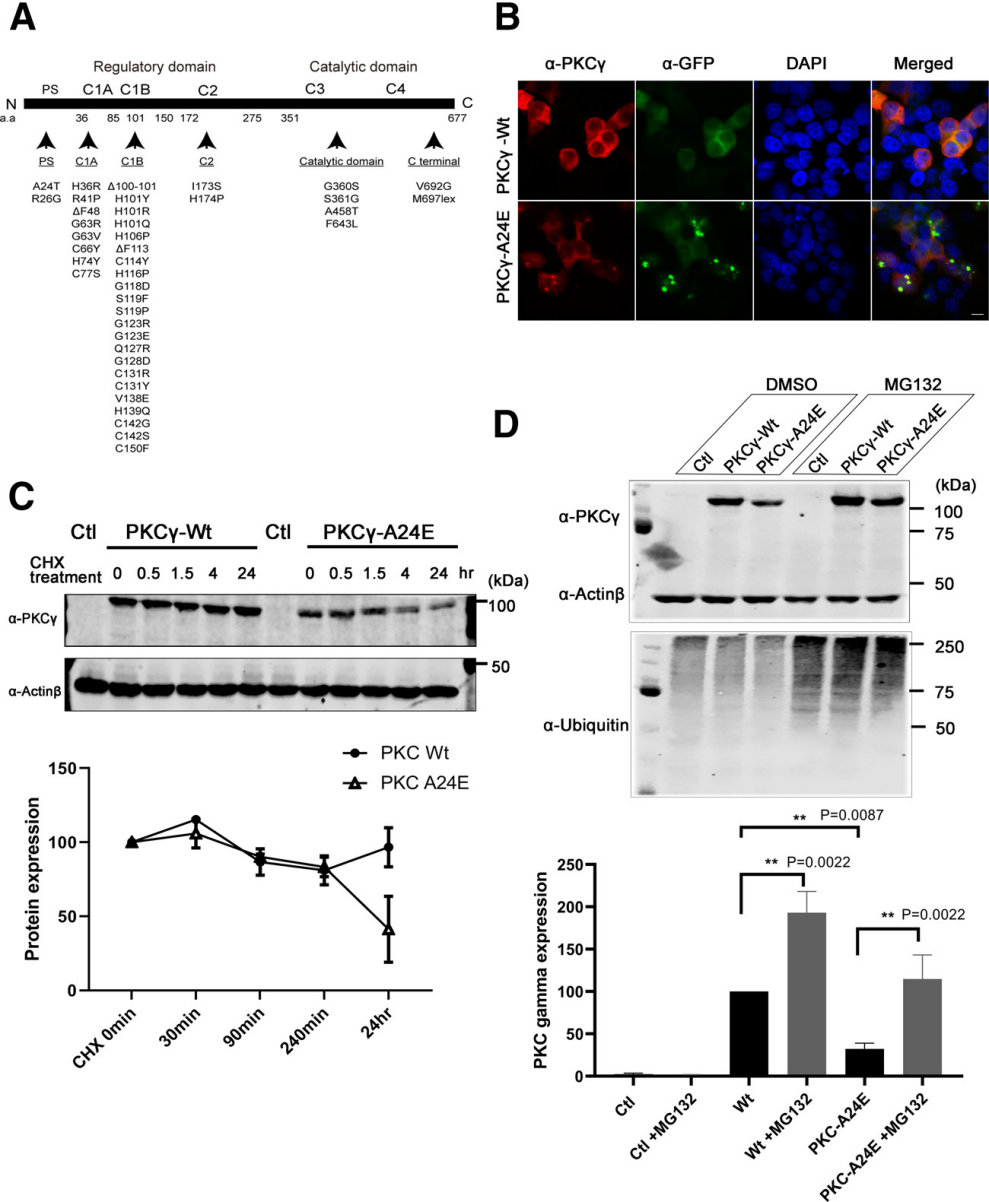
Pseudosubstrate mutant PKCγ protein is unstable and shows aggregation. ***A***, Illustrations of PKCγ protein domain mutations and deletions found in SCA14 families. Most mutations are found in the C1B domain. ***B***, The 5 µg of GFP-Control plasmid, GFP-PKCγ-Wt, or GFP-PKCγ-A24E was transfected to the HeLa cells; and after 24 h, cells were fixed with PFA following the immunostaining. GFP-PKCγ-A24E showed aggregation and accumulated in HELA cells. Images were acquired with confocal microscopy (Carl Zeiss, LSM700) using a Plan-Apochromat 100×/1.3 Oil DIC M27 objective (Carl Zeiss). Scale bar, 10 µm. ***C***, Pseudosubstrate domain mutant PKCγ is unstable and is degraded after 35 µg/ml cycloheximide treatment. After 48 h transfection, cycloheximide was applied to the cells. Samples were collected at 0 min, 30 min, 90 min, 240 min, and 24 h. Twenty-four hours after cycloheximide treatment, the PKCγ protein expression level is GFP-PKCγ-Wt = 96.68% and GFP-PKCγ-A24E = 41.27% compared with each starting point, respectively. ***D***, Degradation of pseudosubstrate domain mutant PKCγ occurs via the proteasome pathway. Twenty-four hours after 5 μm of proteasome inhibitor MG132 treatment, HEK293T cells show more ubiquitinated proteins, and this treatment rescued GFP-PKCγ-A24E protein levels (GFP-PKCγ-Wt = 100.0%; GFP-PKCγ-Wt + MG132 = 192.90% ± 25.35; GFP-PKCγ-A24E = 32.26% ± 6.75; GFP-PKCγ-A24E + MG132 = 114.60% ± 28.54; *n* = 6). Protein expression was analyzed using the two-tailed Mann–Whitney test (GFP-PKCγ-Wt vs GFP-PKCγ-A24E, *p* = 0.0022, GFP-PKCγ-Wt vs GFP-PKCγ-Wt + MG132, *p* = 0.0022; GFP-PKCγ-A24E vs GFP-PKCγ-A24E + MG132, *p* = 0.0087).

There are now >40 different missense mutations or deletions which have been found in human SCA14 patients (Chelban et al., 2018; Wong et al., 2018) (Fig. 1*A*). We have previously reported that, within these mutations, PKCγ-S361G has increased kinase activity resulting in abnormal Purkinje cell development (Ji et al., 2014; Shimobayashi and Kapfhammer, 2018). The PKCγ-S361G mutation is located in the kinase domain and thus not subject to regulation of PKC activity within the cell. We now aimed to generate a constitutive active form of PKCγ by a mutation in the regulatory parts of the molecule. From PKCα, it is known that the substitution of the conserved alanine residue in the pseudosubstrate site of PKCα with a charged glutamic acid residue (E25) causes a reduction in the affinity of this sequence on the catalytic domain leading to the activated conformation of the PKCα protein (Pears et al., 1990). The pseudosubstrate domain is well conserved and exactly the same amino acid sequence from amino acids 22-29 from PKCα is found in PKCγ with the alanine residue being in position 24. We first introduced the single point mutation on alanine 24 changing it to glutamic acid, called PKCγ-A24E. Then GFP-PKCγ-A24E was transfected to HeLa cells or HEK293T cells to observe the protein expression. As shown in Figure 1*B*, GFP-PKCγ-A24E had a strong tendency for aggregation compared with GFP-PKCγ-Wt, and the amount of protein expression was markedly reduced (GFP-PKCγ-Wt = 100% vs GFP-PKCγ-A24E = 29.7%; Fig. 1*C*, at 0 min [Starting point]). In order to see the protein half-life and degradation, we applied the translation inhibitor cycloheximide. Twenty-four hours after cycloheximide treatment, GFP-PKCγ-Wt protein was still present in normal amounts (96.68% of the original GFP-PKCγ-Wt expression), whereas GFP-PKCγ-A24E was reduced down to 41.27% of the original GFP-PKCγ-A24E expression (Fig. 1*C*), indicating that the reduced amounts of GFP-PKCγ-A24E protein are because of increased degradation. As it is known that PKC protein family is mainly degraded via ubiquitin proteasome pathway (Wang et al., 2016), we used the proteasome inhibitor MG132 to block the ubiquitin proteasome degradation. Twenty-four hours after MG132 treatment, reduced GFP-PKCγ-A24E protein level was rescued (PKCγ-Wt = 100.0%; PKCγ-Wt + MG132 = 192.9%; PKCγ-A24E = 32.26%; PKCγ-A24E + MG132 = 114.6%; *n* = 6), indicating that GFP-PKCγ-A24E is unstable in cells and at least partially targeted for degradation via the proteasome pathway (Fig. 1*D*).

### A24E overexpression in Purkinje cells induces a strong reduction of dendritic development

**Figure 2.**
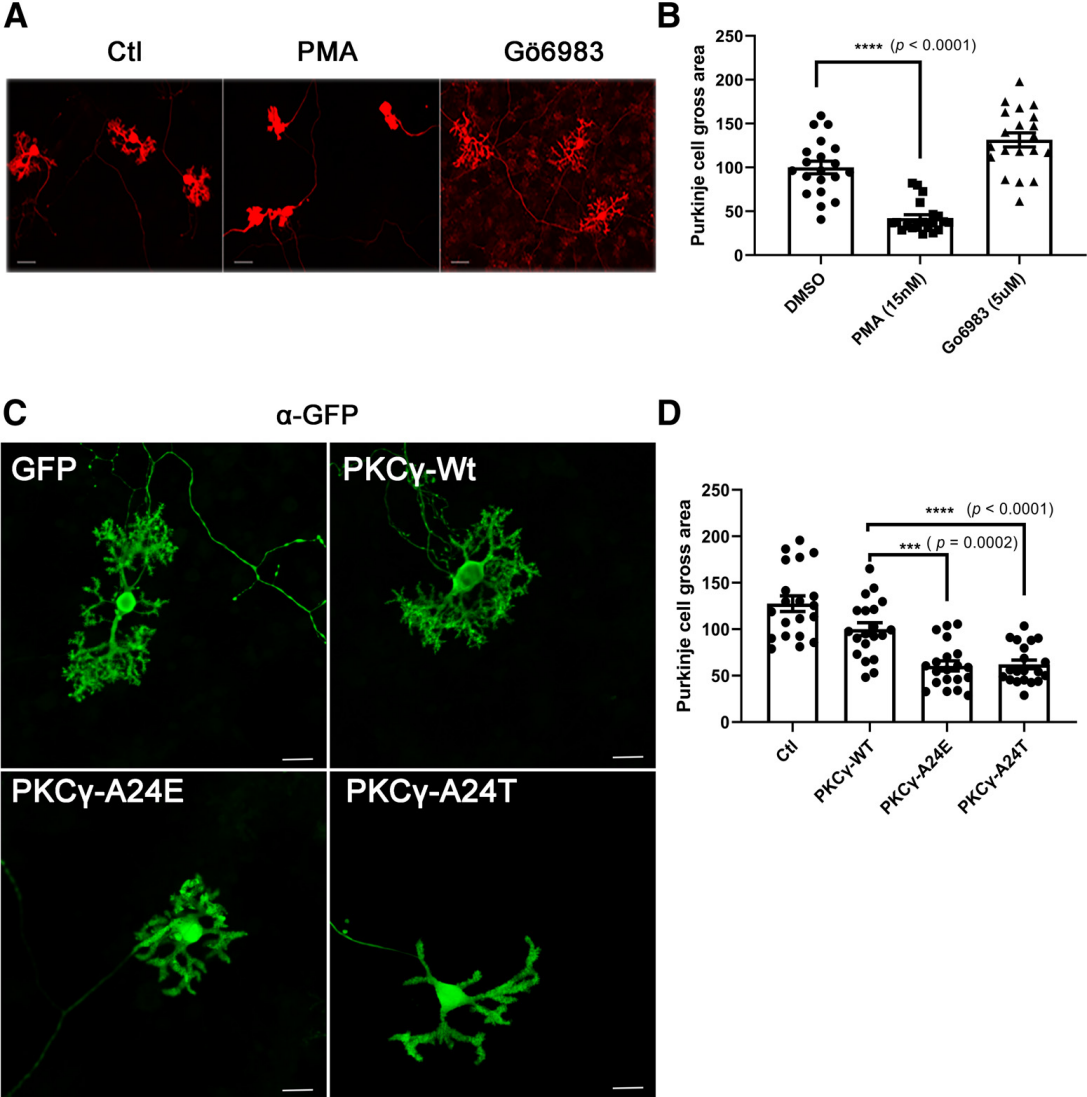
Immunofluorescence staining of Purkinje cells transfected with PKCγ pseudosubstrate domain mutants. ***A***, The morphology of Purkinje cells was analyzed after 2 weeks in dissociated cerebellar culture. Purkinje cells treated with 15 nm PMA for 1 week show small dendrites. Scale bar, 50 µm. ***B***, Differences between control and PMA-treated Purkinje cell size were analyzed using the two-tailed Mann–Whitney test (Wt + DMSO = 100.0% ± 7.15; Wt + PMA = 42.21% ± 3.92; Wt + Gö6983 = 131.5% ± 7.98; for DMSO vs PMA, *p* < 0.0001). Data are mean ± SEM of 20 Purkinje cells. ***C***, Purkinje cells transfected with pseudosubstrate domain mutations show inhibition of dendritic growth with anti-GFP staining. Scale bar, 20 µm. Images were acquired with a confocal microscope (Carl Zeiss, LSM710) equipped with solid state lasers using a LD Plan-Neofluar objective (Carl Zeiss). ***D***, Quantification of the Purkinje cell area. The dendritic tree size of Purkinje transfected with PKCγ pseudosubstrate domain mutations GFP-PKCγ-A24E or GFP-PKCγ-A24T was strongly reduced compared with that of GFP-PKCγ-Wt (GFP-PKCγ-Wt = 100.0% ± 6.92; GFP-PKCγ-A24E = 62.16% ± 4.68; GFP-PKCγ-A24T = 60.53% ± 5.35). Each Purkinje cell area was measured with ImageJ and analyzed with GraphPad Prism. Differences between GFP-PKCγ-Wt and GFP-PKCγ-A24E or GFP-PKCγ-Wt and GFP-PKCγ-A24T were analyzed using the two-tailed Mann–Whitney test (GFP-PKCγ-A24E, *p* = 0.0002 and GFP-PKCγ-A24T, *p* < 0.0001). Data are mean ± SEM of 20 Purkinje cells.

We previously reported that PKC activation negatively regulates Purkinje cell dendritic development (Ji et al., 2014). PKC activator-treated Purkinje cells show compromised dendrites, whereas PKC inhibitor-treated Purkinje cells show dendrites with increased branching (Shimobayashi and Kapfhammer, 2018) (Fig. 2*A*,*B*). Therefore, Purkinje cell dendritic development is an indicator of biological PKC kinase activity. We then tested whether mutant PKCγ-A24E would affect Purkinje cell dendritic development in dissociated cerebellar culture. Purkinje cells were transfected with PKCγ carrying either the A24E mutation or the A24T mutation, which was found in human SCA14 patients (Chelban et al., 2018). The L7-based plasmids yielded a Purkinje cell-specific expression of the mutated proteins. In order to confirm the transfection and expression of Purkinje cells easily, Wt and mutant PKCγ-GFP fusion constructs were generated, and Purkinje cells expressed fusion proteins with PKCγ-Wt, PKCγ-A24E, or PKCγ-A24T fused to GFP. Purkinje cells transfected with GFP-PKCγ-A24E or GFP-PKCγ-A24T only developed small dendritic trees with few side branches (Fig. 2*C*). This morphology is identical to that of PKC activator-treated Purkinje cells (Fig. 2*A*). Statistical analysis showed a significant reduction of the gross area of A24E or A24T mutant PKCγ-transfected Purkinje cells compared with that of GFP-transfected Purkinje cells or PKCγ-Wt-transfected Purkinje cells (GFP-PKCγ-Wt = 100.0 ± 6.92%, *n* = 20; GFP-PKCγ-A24E = 62.16 ± 4.68%, *n* = 20, *p* = 0.0002; GFP-PKCγ-A24T =60.50 ± 5.35%, *n* = 20, *p* < 0.0001) (Fig. 2*D*). These results indicate that two pseudosubstrate domain mutations negatively regulate dendritic growth.

### *PKC*γ*-A24E* mice show less PKCγ protein compared with control

**Figure 3.**
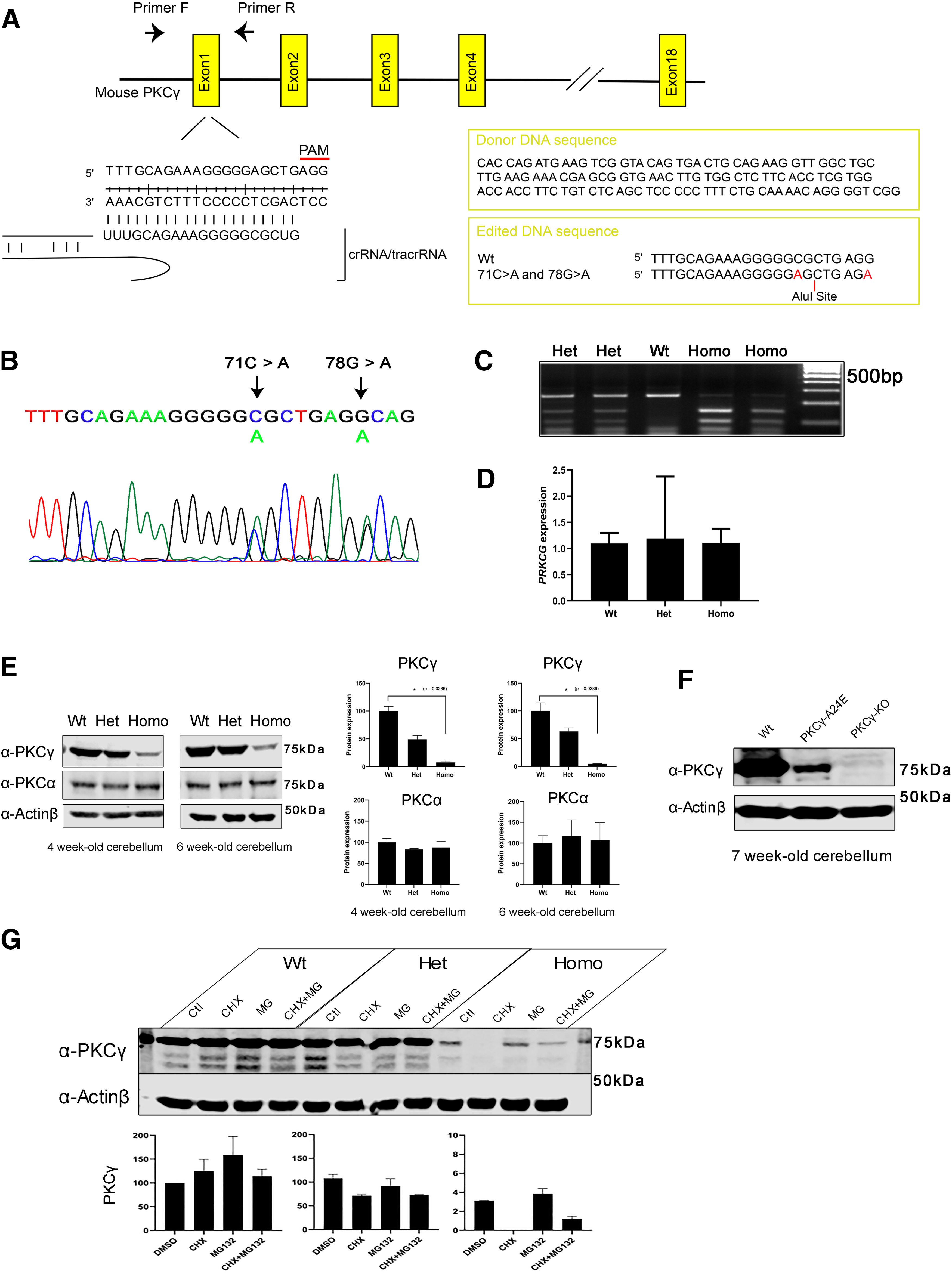
Generation and characterization of the *PKC*γ*-A24E* mouse. ***A***, Target sequence in the *PRKCG* gene (chromosome 7, 1.93 cM) for producing point mutated (c. 71C > A; p. Ala 24 Glu) *PRKCG* with Cas9/CRISPR engineering system. To prevent donor DNA cleavage, the PAM sequence mutation was also introduced (78G > A), which does not give a change in amino acids. ***B***, Mutations were validated with sequencing. The figure shows Het *PKC*γ*-A24E* sequence. ***C***, To identify the genotypes, PCR was performed followed by Alu1 (BioLabs, R0137S) digestion for 1 h, which digests only Ala 24 Glu mutated DNA. Wt shows a single band (250 bp), Het *PKC*γ*-A24E* shows a Wt band and two digested bands (250, 150, and 100 bp), and Homo *PKC*γ*-A24E* shows only two digested bands (150 and 100 bp). ***D***, qPCR using 5-week-old cerebellar samples from each genotype. Data show that all three genotypes express PKCγ mRNA. ***E***, Western blot analysis of total PKCγ protein in the cerebellum from the three genotypes (*n* = 4). PKCγ protein degradation in *PKC*γ*-A24E* mice was observed (4-week-old: Wt = 100.0 ± 8.34%, *n* = 4; Het = 49.12 ± 6.80%, *n* = 4; Homo = 7.519 ± 2.48%, *p* = 0.0286, *n* = 4; 6-week-old: Wt = 100.0 ± 14.56%, *n* = 4; Het = 63.09 ± 6.39%, *n* = 4; Homo = 4.674 ± 0.40%, *p* = 0.0286, *n* = 4), whereas PKCα protein expression did not change in all genotypes (4-week-old: Wt = 100.0 ± 9.30%, *n* = 4; Het = 83.13 ± 2.09%, *n* = 4; Homo = 87.67 ± 14.1%, *n* = 4; 6-week-old: Wt = 100.0 ± 8.94%, *n* = 4; Het = 117.4 ± 19.3%, *n* = 4; Homo = 106.8 ± 21.1%, *n* = 4). The total expression levels of PKCγ and PKCα were normalized to the Actinβ expression level, and the expression levels of PKCγ and PKCα in Wt are shown as 100%. Data are mean ± SEM (*n* = 4, the statistical analysis showed no significance). Differences between Wt and Homo *PKC*γ*-A24E* were analyzed using the two-tailed Mann–Whitney test. ***F***, Western blot analysis of total PKCγ protein in cerebellum from 7-week-old Wt, *PKC*γ*-A24E*, and *PKC*γ *KO* mice. PKCγ protein expression in Homo *PKC*γ*-A24E* mouse is confirmed via Western blot with long exposure, whereas *PKC*γ *KO* mouse shows no PKCγ protein expression (Wt = 100.0%, *n* = 4; Homo *PKC*γ*-A24E* = 2.088%, *n* = 4; *PKC*γ *KO* = 0.067%, *n* = 4). ***G***, Organotypic slice culture from Wt, Het, and Homo *PKC*γ*-A24E* mice treated with 50 µg/ml cycloheximide or 10 μm MG132 or both at DIV7; protein was extracted at DIV8. Western blot data show that PKCγ protein expression is strongly reduced in *PKC*γ*-A24E* mice after cycloheximide treatment, whereas MG132 treatment rescued protein degradation in *PKC*γ*-A24E* mice. The total expression level of PKCγ was normalized to Actinβ expression level and DMSO-treated Wt is shown as 100.0% (Wt + DMSO = 100.0%; Wt + CHX = 124.9%; Wt + MG132 = 159.0%; Wt + MG132 + CHX = 114.2%; Het + DMSO = 107.8%; Het + CHX = 71.3%; Het + MG132 = 91.7%; Het + MG132 + CHX = 73.0%; Homo + DMSO = 3.1%; Homo + CHX = 0.03%; Homo + MG132 = 3.8%; Homo + MG132 + CHX = 1.2%, *n* = 2).

We generated a *PKC*γ*-A24E* knock-in mouse with CRISPR/Cas9-mediated genome editing methods (Menke, 2013) to observe the mutated PKCγ function in Purkinje cells. We introduced the point mutations (c. 71C > A; p. Ala 24 Glu, c. 78G > A; p. Arg 26 Arg) in the *PRKCG* gene (Fig. 3*A–C*). A R26R mutation in the PAM sequence which does not change the amino acids was also introduced to prevent the donor DNA from being a suitable target for Cas9 cleavage. After identification of mutant founders with PCR and sequencing, we characterized the new knock-in mouse called *PKC*γ*-A24E* mouse.

The Het and Homo *PKC*γ*-A24E* mice had normal survival and growth compared with Wt littermates (data not shown). The PKCγ protein expression level in cerebellum in Homo *PKC*γ*-A24E* mouse was drastically reduced (Fig. 3*E*) at all developmental stages (4-week-old: Wt = 100.0 ± 8.34%, *n* = 4; Het = 49.12 ± 6.80%, *n* = 4; Homo = 7.519 ± 2.48%, *p* = 0.0286, *n* = 4; 6-week-old: Wt = 100.0 ± 14.56%, *n* = 4; Het = 63.09 ± 6.39%, *n* = 4; Homo = 4.674 ± 0.40%, *p* = 0.0286, *n* = 4). PKCγ protein in the *PKC*γ*-A24E* mice was strongly reduced but still present (Fig. 3*F*) compared with that of the *PKC*γ *KO* mouse. The expression of PKCα, another classical PKC isoform expressed in Purkinje cells, was unchanged in all genotypes (Fig. 3*E*). mRNA expression in Het and Homo was comparable to that of Wt littermates (Fig. 3*D*), indicating that translation of A24E mutant PKCγ is normal. Cycloheximide trace assay showed increased protein degradation in Homo *PKC*γ*-A24E* mice, whereas proteasome inhibitor MG132 treatment for 24 h partially rescued the reduced amount of PKCγ expression (Fig. 3*G*). These data agree with the findings in cell transfection assays (Fig. 1) and confirm that mutant *PKC*γ*-A24E* protein is rapidly targeted for degradation. In Purkinje cells from *PKC*γ*-A24E* mice, we did not observe aggregation of mutant PKCγ-A24E protein as seen with overexpression in cell lines.

### Increased PKC kinase activity in *PKC*γ*-A24E* mice despite reduced protein levels

**Figure 4.**
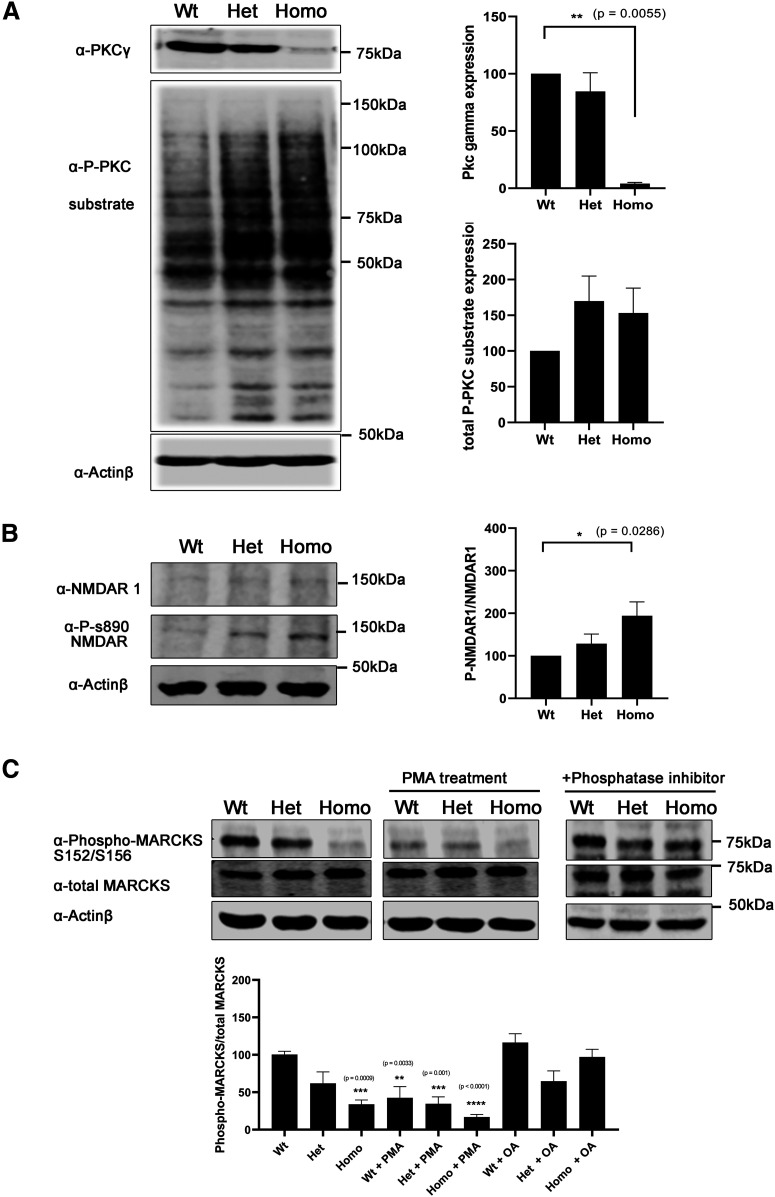
*PKC*γ*-A24E* mouse shows highly PKC kinase activity. ***A***, Western blot analysis of total PKCγ protein and phospho-PKC substrates from organotypic slice cultures. Phosphorylation of PKC substrates was increased in Het and Homo PKCγ-A24E mice. ***B***, Western blot analysis normalized to actin shows PKCγ protein reduction in *PKC*γ*-A24E* mice, but phospho-PKC substrate is upregulated in *PKC*γ*-A24E* mice. NMDAR-S890 is known to be phosphorylated by PKCγ. Phospho-NMDAR-S890 protein expression is normalized to total NMDAR-N1 protein expression. This phosphorylation is increased in *PKC*γ*-A24E* mice. The data were normalized to Wt as 100% from three independent experiments. ***C***, Phospho-MARCKS is a known substrate of PKC. Phospho-MARCKS S152/S156 compared with total MARCK was decreased in *PKC*γ*-A24E* mice and PMA-treated organotypic slice cultures from Wt mice (Wt = 100.4 ± 4.37%, *n* = 4; Het = 61.92 ± 15.25%, *n* = 4; Homo = 33.98 ± 5.83%, *p* = 0.0009, *n* = 4; Wt + PMA = 42.54 ± 15.4%, *p* = 0.0033, *n* = 4; Het + PMA = 34.58 ± 9.39%, *p* = 0.0010, *n* = 4; Homo + PMA = 16.89 ± 3.557%, *p* < 0.0001, *n* = 4). With added phosphatase inhibitor, the phospho-MARCKS reduction was partially rescued (Wt = 116.5 ± 11.8%, *n* = 3; Het = 64.68 ± 13.8%, *n* = 3; Homo = 97.15 ± 10.3%, *n* = 3). The two-tailed Mann–Whitney test was used to analyze the difference between each group, and Wt without any treatment is shown as 100%. Data are mean ± SEM.

As the mutation in the pseudosubstrate domain is supposed to keep the protein in a constantly active open conformation, we studied whether the overall PKC activity in cerebellar slice cultures of *PKC*γ*-A24E* mice was reduced or increased. We used a general anti-phospho-serine PKC substrate antibody to see whether the phosphorylation of PKC substrates was increased or not and found that both Het and Homo *PKC*γ*-A24E* mice had increased PKC kinase activity compared with Wt littermate controls (Fig. 4*A*). We also studied the phosphorylation of a known PKCγ target protein, the NMDAR, which is composed of a heterodimer of at least one NR1 and one NR2A-D subunit. The NR1 subunit can be phosphorylated by PKCγ at Ser890 (Sánchez-Pérez and Felipo, 2005). Using a phospho-specific antibody, we found that phosphorylation at this site was markedly increased both in Het and Homo PKCγ-A24E mice (Fig. 4*B*). MARCKS is also a known phosphorylation target of PKC in many cells. Surprisingly, phospho-MARCKS protein expression compared with total MARCKS expression was strongly decreased in the *PKC*γ*-A24E* mouse. A similar reduction was found in Wt slice cultures treated with PMA (Wt = 100.4 ± 4.37%, *n* = 4; Het = 61.92 ± 15.25%, *n* = 4; Homo = 33.98 ± 5.83%, *p* = 0.0009, *n* = 4; Wt + PMA = 42.54 ± 15.4%, *p* = 0.0033, *n* = 4; Het + PMA = 34.58 ± 9.39%, *p* = 0.0010, *n* = 4; Homo + PMA = 16.89 ± 3.557%, *p* < 0.0001, *n* = 4), suggesting that phospho-MARCKS protein is more dephosphorylated by phosphatases under the PKCγ activation condition. This dephosphorylation could be mostly prevented by the use of the phosphatase inhibitor okadaic acid (Fig. 4*C*).

### Altered morphology of Purkinje cells from PKCγ-A24E mice *in vitro*

**Figure 5.**
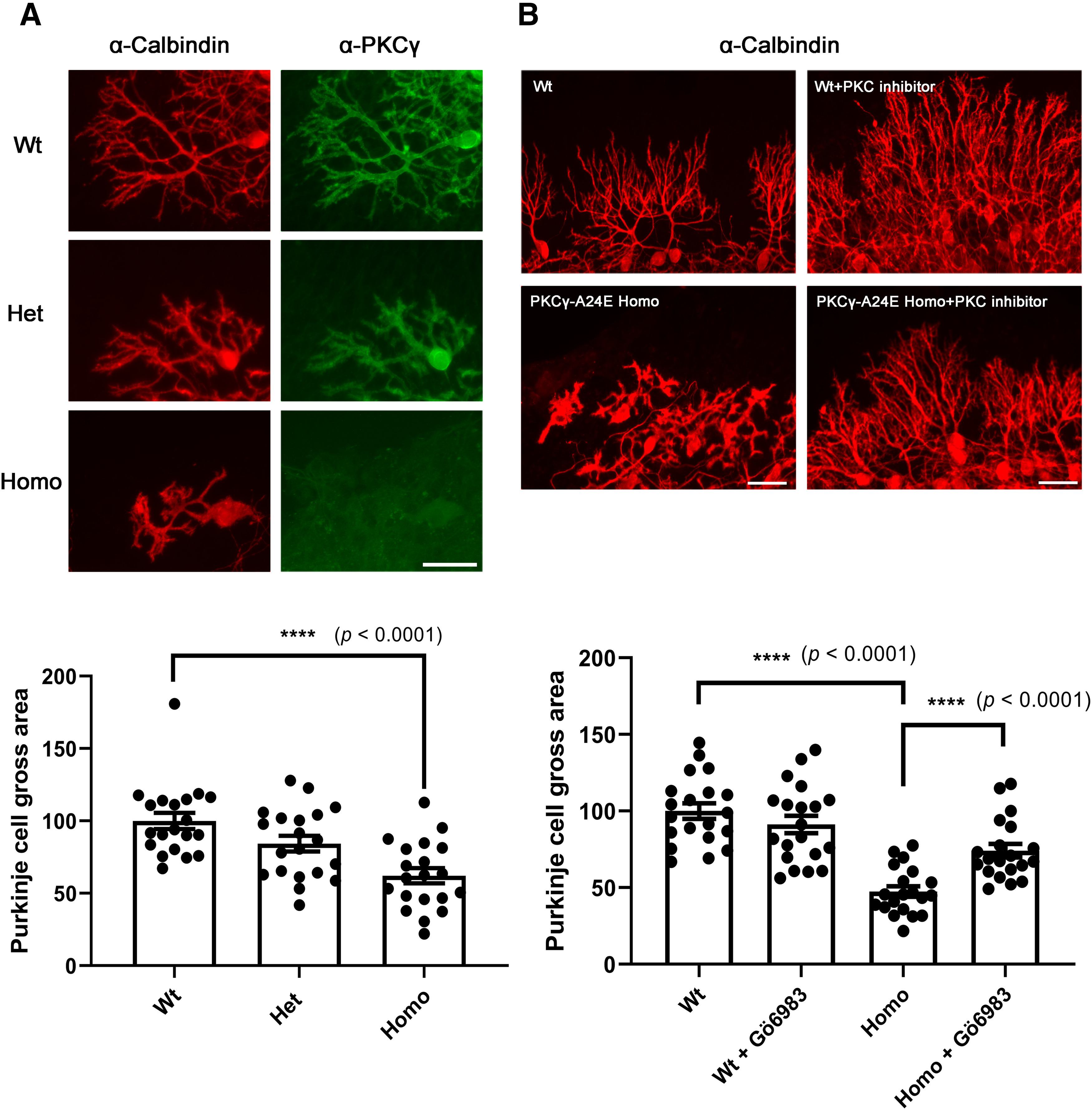
Altered morphology of Purkinje cells from *PKC*γ*-A24E* mice in organotypic cerebellar slice culture that is rescued by PKC inhibitor. ***A***, Purkinje cells in organotypic slice cultures from each genotypes are shown. Anti-calbindin staining showing all Purkinje cells, and PKCγ expression was strongly reduced in *PKC*γ*-A24E* mice with anti-PKCγ immunostaining. Scale bar, 50 µm. Each Purkinje cell area was measured with ImageJ and analyzed with GraphPad Prism. Differences between PKCγ-Wt and PKCγ-A24E were analyzed using the two-tailed Mann–Whitney test (Wt = 100.0 ± 5.64%; Het = 84.29 ± 5.40%, *p* = 0.0718 and Homo = 62.14 ± 5.23%, *p* < 0.0001). Data are mean ± SEM of 20 Purkinje cells. ***B***, Purkinje cells from organotypic slice cultures at DIV 7 with or without PKC inhibitor (Gö6983) treatment. For control, the same volume of DMSO was added, and 10 μm Gö6983 was added at DIV3. Control Purkinje cells show elaborate branched dendrites, whereas Purkinje cells from *PKC*γ*-A24E* mice have reduced dendritic growth, which was rescued by PKC inhibitor (Gö6983) treatment. The dendritic area of Purkinje cells was measured with ImageJ, and the analysis shows that PKC inhibitor treatment could rescue the dendritic tree size (Purkinje cells area Wt = 100.0 ± 5.00%; Homo PKCγ-A24E = 47.50 ± 3.42%; Homo PKCγ-A24E + Gö6983 = 74.11 ± 4.40%). Differences between PKCγ-A24E and Gö6983-treated PKCγ-A24E were analyzed using the two-tailed Mann–Whitney test. (*p* < 0.0001). The number of measured cells was 20 for all experiments.

We studied the dendritic morphology of Purkinje cells from *PKC*γ*-A24E* mice. In organotypic slice culture, the dendritic expansion of Purkinje cells from PKCγ-A24E mice was severely impaired and the cells developed only short thickened dendrites similar to those found after PMA treatment (Ji et al., 2014). This was most evident in Homo *PKC*γ*-A24E* mice, but also noticeable in Het *PKC*γ*-A24E* mice (Fig. 5*A*). Statistical analysis showed that the size of Purkinje cells from Homo PKCγ-A24E mice (62.1 ± 5.23%, *p* < 0.0001) is significantly smaller than that of Wt (100 ± 5.64%). Purkinje cell size of Het is also smaller than that of Wt (Het, 84.2 ± 5.40%, *p* = 0.0718), but the difference did not reach statistical significance (values are shown as percentage of Wt [100%] and are the mean ± SEM of 20 Purkinje cells).

As this change in morphology is supposed to be because of increased PKC activity, we checked whether a PKC inhibitor might rescue the dendritic morphology in PKCγ-A24E mice. We applied the PKC inhibitor Gö6983 to cerebellar slice cultures during the culture period (Fig. 5*B*). Purkinje cells from Homo PKCγ-A24E mice had longer dendrites with more branches after PKC inhibitor treatment, showing a significant rescue (Purkinje cells area Wt = 100.0 ± 5.00%, *n* = 20; Homo PKCγ-A24E = 47.50 ± 3.42%, *n* = 20; Homo PKCγ-A24E + Gö6983 = 74.11 ± 4.40%, *n* = 20), indicating that the reduction in dendritic expansion of Purkinje cells from PKCγ-A24E mice is caused by increased PKC activity (Wt vs Homo PKCγ-A24E, *p* < 0.0001; Homo PKCγ-A24E vs Homo PKCγ-A24E + Gö6983, *p* < 0.0001).

### Altered CF innervation and Purkinje cell morphology in *PKC*γ*-A24E* mice

**Figure 6.**
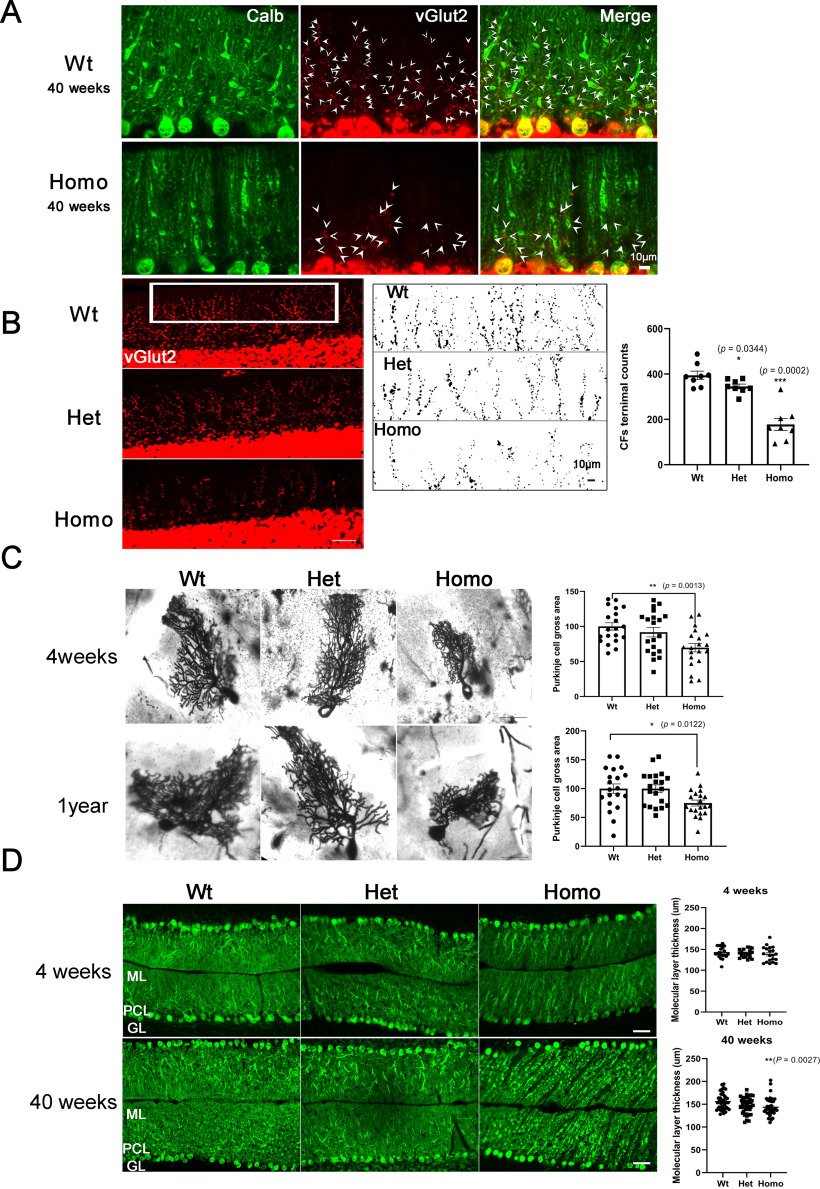
Altered CF innervation and Purkinje cell morphology *in vivo* in *PKC*γ*-A24E* mice. ***A***, Anti-Calbindin D28K antibody staining shows Purkinje cells, and anti-vGlut2 staining shows CF terminals on Purkinje cell dendrites from 40-week-old Wt and Homo *PKC*γ*-A24E* mice. White arrowheads indicate CF terminals. Scale bar, 10 µm. ***B***, The CF terminals on Purkinje cells were analyzed with anti-vGlut2 immunoreactivity. Scale bar, 50 µm. The CF terminals in the molecular layer were counted by ImageJ. Quantification of the number of terminal puncta on Purkinje cell branchlets in distal molecular layer in a 284 μm × 75 μm rectangle (vGlut2-positive puncta Wt = 394.4 ± 18.14; Het = 345.1 ± 10.61, *p* = 0.0344; Homo = 177.6 ± 26.62, *p* = 0.0002). Data are mean ± SEM of 8 samples each. Scale bar, 10 µm. ***C***, Bright field microscopy analysis of Golgi-Cox staining from 4-week-old and 1-year-old Wt, Het, and Homo *PKC*γ*-A24E* mice shows significantly reduced size of Purkinje cells in Homo *PKC*γ*-A24E* mice. Purkinje cell size from 4-week-old (Wt = 100.0 ± 5.36%; Het PKCγ-A24E = 91.85 ± 6.81%; Homo PKCγ-A24E = 69.74 ± 6.07%) and Purkinje cell size from 1-year-old (Wt = 100.0 ± 8.17%; Het *PKC*γ*-A24E* = 99.85 ± 6.51%; Homo *PKC*γ*-A24E* = 74.91 ± 5.14%) were analyzed using the two-tailed Mann–Whitney test (4-week-old, *p* = 0.0013, 1-year-old, *p* = 0.0122). Scale bar, 50 µm. The number of measured cells was 20 for all experiments. ***D***, Calbindin D-28 immunohistochemistry staining of cerebellar cryosections (20 µm) from 4-week-old to 40-week-old Wt, Het PKCγ-A24E, and Homo *PKC*γ*-A24E* mice. ML, Molecular layer; PCL, Purkinje cell layer; GL, granule cell layer. Scale bar, 50 µm. The width of the molecular layer is slightly decreased in lobule VII of 40-week-old Homo *PKC*γ*-A24E* mice (*p* = 0.0027).

Synapse formation on Purkinje cells from parallel fibers (PFs) and CFs is well controlled during postnatal developmental and is crucial for cerebellar function (Kano et al., 1995). It is known that activation of the mGluR1-PLCβ-PKCγ signaling pathway is involved in the regulation of this synapse formation: the initially overlapping CFs become reduced by CF elimination during the first three postnatal weeks resulting in a one CF to one Purkinje cell relationship (Kano et al., 2018), which is essential for the establishment of the precise neuronal circuit in the cerebellum (De Zeeuw et al., 1998; Shuvaev et al., 2011; Ichikawa et al., 2016). In PKCγ-deficient mice, multiple CF innervation is disturbed (Kano et al., 1995) while mutant PKCγ transgenic mice showed a reduced innervation of Purkinje cells by CF terminals (Trzesniewski et al., 2019). PKCγ is also involved in the formation of appropriate PF/CF territories (Ichikawa et al., 2016). Together, these findings show that PKCγ signaling is required for proper PF/CF formation and innervation. We studied the CF terminals on Purkinje cells in *PKC*γ*-A24E* mice with the CF marker vGlut2 and compared them with Wt mice. Purkinje cells from Wt mice are innervated by CF on their proximal dendrites covering 70%-90% of the molecular layer. In contrast, CF terminals in *PKC*γ*-A24E* mice have a reduced density in particular on the distal part of the dendrites (Fig. 6*A*). We quantified the difference by counting the number of terminal puncta and found significantly less CF terminals in the molecular layer on the distal part of the dendrites from 40-week-old *PKC*γ*-A24E* mice (Fig. 6*B*). This finding indicates that proper CF innervation is disturbed by the increased PKC activity in *PKC*γ*-A24E* mice. We further studied the morphology of Purkinje cells from *PKC*γ*-A24E* mice *in vivo* using the Golgi-Cox impregnation method because only few Purkinje cells become labeled with this method, which avoids problems with overlapping Purkinje cell dendrites. We found that, in Homo *PKC*γ*-A24E* mice, Purkinje cells had less extended, condensed dendritic trees covering a reduced area (Fig. 6*C*) both in 4-week-old (Purkinje cells area Wt = 100.0 ± 5.36%; Het = 91.85 ± 6.81%; Homo = 69.74 ± 6.07%, *p* = 0.0013; *n* = 20 for each genotype) and 1-year-old mice (Purkinje cells area Wt = 100.0 ± 8.17%; Het = 99.85 ± 6.51%; Homo = 74.91 ± 5.14%, *p* = 0.0122; *n* = 20 for each genotype). This finding is similar to that previously observed in *PKC*γ*-S361G* transgenic mice (Trzesniewski et al., 2019). We also investigated the thickness of the molecular layer where Purkinje cell dendrites extend. The thickness of the molecular layer measured in lobule VII was slightly reduced in 40-week-old Homo *PKC*γ*-A24E* (Fig. 6*D*; Wt, 155.5 ± 2.832 μm; Homo, 143.4 ± 3.137 μm, *p* = 0.0027; *n* = 40 for each genotype). These data indicate that *PKC*γ*-A24E* mice have a reduction of Purkinje cell dendritic tree size and structure *in vivo*. We did not notice an obvious Purkinje cell loss in *PKC*γ*-A24E* mice from 4 to 40 weeks of age.

### *PKC*γ*-A24E* mice are ataxic

**Figure 7.**
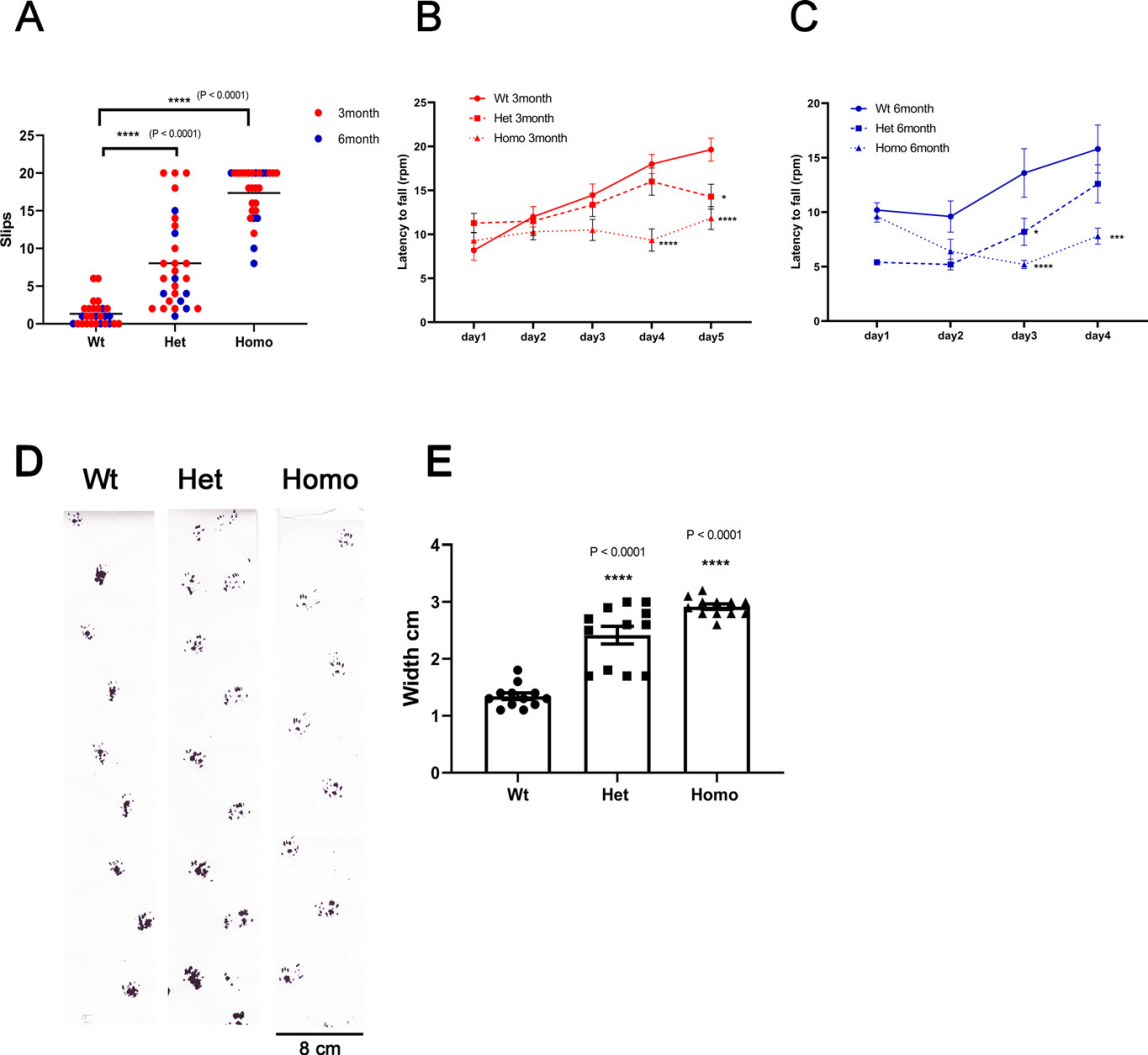
Behavior analysis shows motor deficit and ataxic phenotype in *PKC*γ*-A24E* mice. ***A***, The beam test was done in 3- and 6-month-old mice. Slip count of Wt (1.235 ± 0.271, *n* = 28), Het (9.265 ± 1.079, *n* = 28, *p* < 0.0001), and Homo *PKC*γ*-A24E* mice (17.50 ± 0.056, *n* = 28, *p* < 0.0001). Data are mean ± SEM. Statistical significance of the values of Wt and PKCγ-A24E mice groups was analyzed by two-way ANOVA test with Bonferroni correction: ****p < 0.0001. ***B***, ***C***, Rotarod performance test was evaluated in 3-month-old mice (***B***) and 6-month-old mice (***C***). ***B***, Latency to fall on day 4: Wt = 17.00 ± 2.000 rpm, *n* = 11; Het = 18.00 ± 1.528 rpm, *n* = 14; Homo = 4.333 ± 1.212 rpm, *p* < 0.0001, *n* = 14 and on day 5: Wt = 24.33 ± 1.202 rpm, *n* = 11; Het = 14.67 ± 1.202 rpm, *p* = 0.0317, *n* = 14; Homo = 6.67 ± 0.667 rpm, *p* < 0.0001, *n* = 14. ***C***, Latency to fall on day 3: Wt = 13.60 ± 2.227 rpm; Het = 8.200 ± 1.241 rpm, *p* = 0.0127; Homo = 5.200 ± 0.374 rpm, *p* < 0.0001; *n* = 5 for each genotype and on day 4: Wt = 15.80 ± 2.200 rpm; Het = 12.60 ± 1.749 rpm; Homo = 7.80 ± 0.735 rpm, *p* = 0.0002; *n* = 5 for each genotype. Data are mean ± SEM. Statistical significance of the values of Wt and *PKC*γ*-A24E* mice groups was analyzed by two-way ANOVA test with Bonferroni correction: *p < 0.05; ***p < 0.001; ****p < 0.0001. ***D***, Footprints of 9-month-old Wt and *PKC*γ*-A24E* mice were evaluated for step width. ***E***, Wt = 1.30 ± 0.058 cm, *n* = 12; Het = 2.60 ± 0.154 cm, *n* = 12; Homo = 2.95 ± 0.047 cm, *n* = 12. Data are mean ± SEM. Statistical significance of the values of Wt and Het or Homo *PKC*γ*-A24E* mice groups was analyzed by two-way ANOVA test with Bonferroni correction: ****p < 0.0001.

Ataxia is a prominent phenotype in human SCA14 patients, so we studied the motor coordination of *PKC*γ*-A24E* mice. Subtle motor coordination and balance were assessed with the balance beam test in 3- to 6-month-old mice. After training, mice walked along the 80-cm-long square wooden beam of 8 mm width and lateral slips were counted in two complete and consecutive crossings per day, on 5 consecutive days. The mean number of slips per 80 cm traveled was calculated. Some *PKC*γ*-A24E* mice showed poor performance and could not cross the beam because they fell down or were unable to perform the task. For these mice, we assigned a maximum value of slips = 20. Wt mice could walk the balance beam without or with very few slips (1.235 ± 0.271, *n* = 28), whereas both Het (9.265 ± 1.079, *n* = 28, *p* < 0.0001) and Homo *PKC*γ*-A24E* (17.50 ± 0.056, *n* = 28, *p* < 0.0001) showed marked ataxia both at 3 and 6 months of age (Fig. 7*A*; Movies 1, 2, 3). In the rotarod test, the latency to fall off the accelerating rod was assessed at 4-5 consecutive days. A significant deficit was found in 3-month-old *PKC*γ*-A24E* mice (Fig. 7*B*) on day 4 (Latency to fall: Wt = 17.00 ± 2.000 rpm, *n* = 11; Het = 18.00 ± 1.528 rpm, *n* = 14; Homo = 4.333 ± 1.212 rpm, *p* < 0.0001, *n* = 14) and on day 5 (Latency to fall: Wt = 24.33 ± 1.202 rpm, *n* = 11; Het = 14.67 ± 1.202 rpm, *p* = 0.0317, *n* = 14; Homo = 6.67 ± 0.667 rpm, *p* < 0.0001, *n* = 14). In 6-month-old mice (Fig. 7*C*), a deficit was found on day 3 (Latency to fall: Wt = 13.60 ± 2.227 rpm; Het = 8.200 ± 1.241 rpm, *p* = 0.0127; Homo = 5.200 ± 0.374 rpm, *p* < 0.0001; *n* = 5 for each genotype) and on day 4 (Latency to fall: Wt = 15.80 ± 2.200 rpm; Het = 12.60 ± 1.749 rpm; Homo = 7.80 ± 0.735 rpm, *p* = 0.0002; *n* = 5 for each genotype).

Movie 1. The beam test of Wt. Wt mouse at 25-week-old walking on the 80-cm-long and 8-mm-wide wooden bar. 10.1523/JNEUROSCI.1946-20.2021.video.1

Movie 2. The beam test of Het PKCγ-A24E. Het *PKC*γ*-A24E* mouse at 25-week-old walking on the 80-cm-long and 8-mm-wide wooden bar. 10.1523/JNEUROSCI.1946-20.2021.video.2

Movie 3. The beam test of HomoPKCγ-A24E. Homo *PKC*γ*-A24E* mouse at 25-week-old walking on the 80-cm-long and 8-mm-wide wooden bar. 10.1523/JNEUROSCI.1946-20.2021.video.3

With footprint gait analysis, we calculated the step width. A representative trace image of the walking pattern of 9-month-old Wt, Het, and Homo *PKC*γ*-A24E* mice is shown in Figure 7*D*, highlighting the ataxic gait with increased step width of Het and Homo *PKC*γ*-A24E* mice. The difference in the step width was significant for Het and Homo *PKC*γ*-A24E* mice (Wt = 1.30 ± 0.058 cm, *n* = 12; Het = 2.60 ± 0.154 cm, *n* = 12; Homo = 2.95 ± 0.047 cm, *n* = 12; Fig. 7*D*,*E*). The footprint pattern in both Het and Homo *PKC*γ*-A24E* mice showed clear signs of an ataxic gait.

### Molecular characterization of *PKC*γ*-A24E* mice

**Figure 8.**
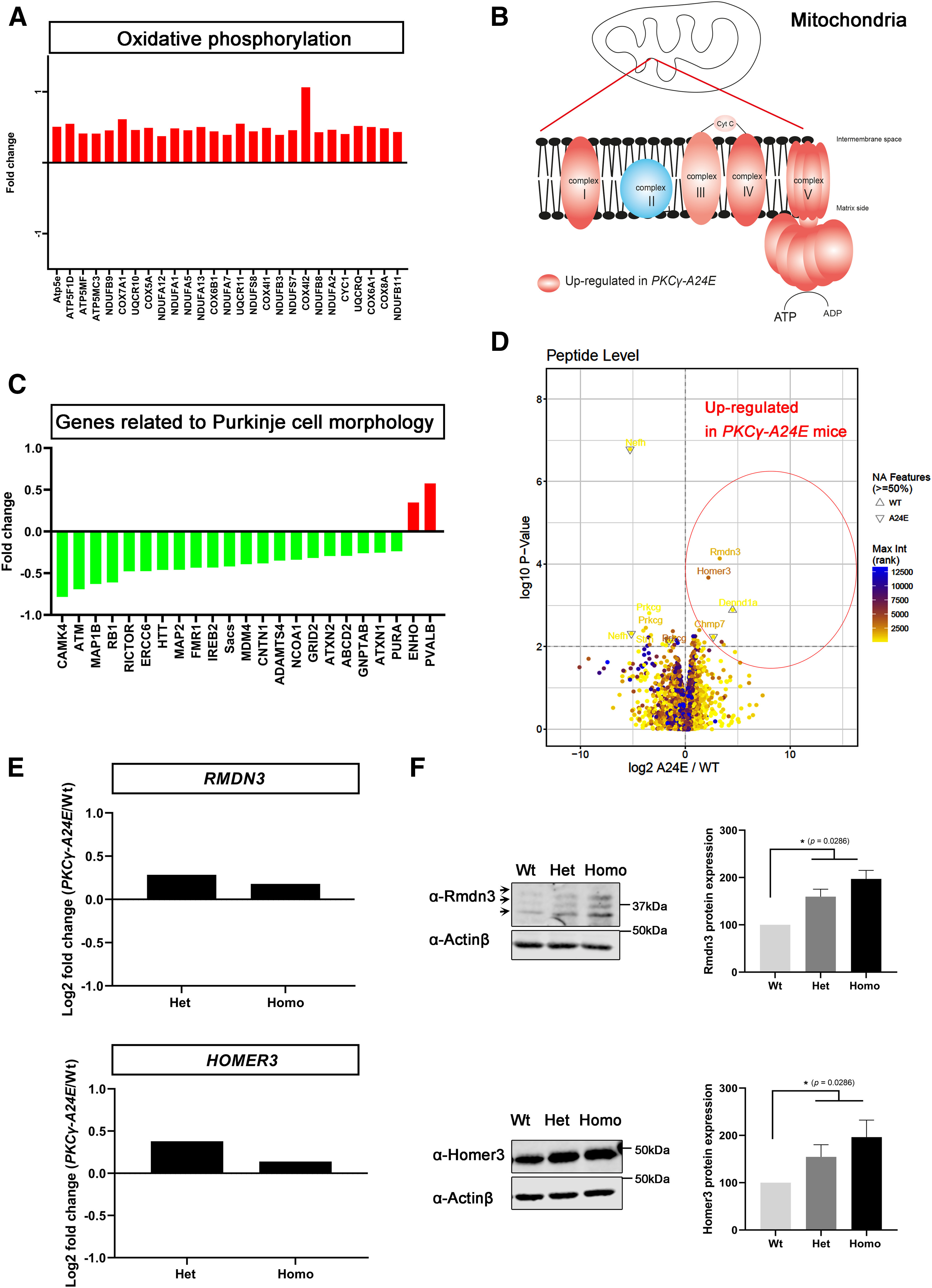
RNA sequence analysis and phosphoproteomics analysis. ***A***, ***B***, Many oxidative phosphorylation-related genes in Complex I, Complex III, Complex IV, and Chemiosmosis are upregulated in Het *PKC*γ*-A24E* mice in RNA sequencing. ***A***, IPA showed that many ubiquinone oxidoreductase subunits and cytochrome *c* oxidase subunits are significantly upregulated in Het *PKC*γ*-A24E* mice. *y* axis is log2 fold change. ***B***, Red complexes are upregulated in Het *PKC*γ*-A24E* mice, which locate in the mitochondrial membrane. ***C***, Network analysis was performed by IPA of gene sets upregulated or downregulated in Het *PKC*γ*-A24E* mice compared with Wt littermates. Green genes represent decreases. Red genes represent increases. Many genes related to Purkinje cell morphology are downregulated in Het *PKC*γ*-A24E* mice. ***D***, Volcano plot of phospho-proteomics analysis of Wt versus Homo *PKC*γ*-A24E* mice (*n* = 3). Lysates from 7-week-old Wt and *PKC*γ*-A24E* mice were subjected to phosphoproteomic analysis by mass spectrometry. Differentially enriched phosphopeptides are shown in the volcano plot. *x* axis is log2 fold change, and *y* axis is *p* value. Rmdn3 and Homer3 are among the proteins with a significantly increased phosphorylation in *PKC*γ*-A24E* mice. ***E***, RNA sequence data show that *Rmdn3 and Homer3* are upregulated in *PKC*γ*-A24E* mice. ***F***, Western blot analysis of Rmdn3 and Homer3 from organotypic slice cultures. Homer3 protein expression (Wt = 100.0%, *n* = 4; Het = 149.7%, *p* = 0.0286, *n* = 4; Homo = 184.2%, *p* = 0.0286, *n* = 4) and Rnmd3 protein expression (Wt = 100.0%, *n* = 4; Het = 167.0%, *p* = 0.0286, *n* = 4; Homo = 206.5%, *p* = 0.0286, *n* = 4) are increased in *PKC*γ*-A24E* mice. Statistical analysis using the two-tailed Mann–Whitney test showing increased protein expression in *PKC*γ*-A24E* mice.

The molecular mechanisms underlying pathogenesis in SCA14 are still unclear. In order to identify changes in gene expression, which might contribute to altered Purkinje cell development and to pathogenesis in mutant PKCγ, we did RNA profiling among Wt, Het, and Homo *PKC*γ*-A24E* mice (*n* = 3 per genotype). For sample preparation, we dissected the molecular layer and Purkinje cell layer of the cerebellum from 5-week-old mice. Total RNA was extracted and subjected to RNA sequence analysis. Interestingly, we found many mitochondrial genes involved in oxidative metabolism to be upregulated in Het *PKC*γ*-A24E* mice according to IPA (QIAGEN) (Fig. 8*A*,*B*; Extended Data Fig. 8-1*A*), indicating that mitochondrial function is altered in Het *PKC*γ*-A24E* mice. On the other hand, molecules that are related to synaptogenesis and glutamate receptor signaling are downregulated (e.g., *HOMER2*↓, *CAMK4*↓, *GRIN2A*↓, *GRIA2*↓, *and GRM5*↓; Extended Data Fig. 8-1*B*). Many genes affecting Purkinje cell development are also downregulated, including *ATM*↓, *ATAXIN*↓, *HTT*↓ *and signaling molecule CAMK4*↓ (Fig. 8*C*). Based on upstream pathway analysis in IPA, we observed a significant change in the mTOR pathway, with *CAB39*↓, *RICTOR*↓, *and STK11*↑, the expression level of many targets of *RICTOR* were altered indicating that the mTOR pathway might one of the targets affected by mutant PKCγ (Extended Data Fig. 8-1*C*). Notably, we identified less significant changes in the expression profile of Homo *PKC*γ*-A24E* mice (Het, 3703 genes; Homo, 206 genes; *p* < 0.01), probably because of compensatory mechanisms. Homo *PKC*γ*-A24E* mice exhibited more upregulated genes related to ephrin receptors (Extended Data Fig. 8-2). It was suggested that activation of the Eph receptors in Purkinje cells may restrict Purkinje cell dendritic spine formation (Cesa et al., 2011; Heintz et al., 2016) and Eph receptors might also be involved in Alzheimer's disease and amyotrophic lateral sclerosis (Yang et al., 2018). The expression of some ionotropic glutamate receptor subunits was also altered (*GRIN2A*↓, *GRID1*↑, *GRIK4*↑, *GRIA1*↑, *GRIK3*↑) in Homo *PKC*γ*-A24E* mice (Extended Data Fig. 8-2); whereas in Het *PKC*γ*-A24E* mice, glutamate receptor signaling was downregulated (*GRIN2A*↓, *GRIA2*↓, *GRM5*↓). In both Het and Homo *PKC*γ*-A24E* mice, GRIN2A was significantly downregulated.

10.1523/JNEUROSCI.1946-20.2021.f8-1 Figure 8-1 Summary of the significantly changed genes in Het PKCγ-A24E mice (symbol, gene name, fold changes, *p* values and locations). (A) Many mitochondrial function related genes are upregulated in Het PKCγ-A24E mice. (B) Summary of the significantly changed genes related to glutamate receptor signaling pathway in Het PKCγ-A24E mice. (C) Summary of the significantly changed genes related to RICTOR signaling pathway in Het PKCγ-A24E mice in RNA sequeincing. Download Figure 8-1, DOCX file.

10.1523/JNEUROSCI.1946-20.2021.f8-2 Figure 8-2 Summary of the significantly changed genes in Homo PKCγ-A24E mice (symbol, gene name, fold changes, *p* values and locations). (A) Summary of the significantly changed genes related to Ehprin receptor signaling pathways in Homo PKCγ-A24E mice. (B) Summary of the significantly changed genes related to glutamate receptor signaling pathway in Homo PKCγ-A24E mice in RNA sequeincing. Download Figure 8-2, DOCX file.

We have also profiled protein phosphorylation by using phospho-proteomics, which might provide valuable insight into the mutant PKCγ signaling. The abundance of 105 phosphopeptides was significantly upregulated while 69 phosphopeptides were significantly downregulated in Homo *PKC*γ*-A24E* mice (*p* < 0.05) (Table 2; Extended Data Fig. 8-3). The volcano plot data show proteins with increased phosphorylation in Homo *PKC*γ*-A24E* mice to the right side and with less phosphorylation to the left side (Fig. 8*D*). The data show that Rmdn3 (also known as *PTPIP51*) and Homer3 are highly phosphorylated in Homo *PKC*γ*-A24E* mice (Fig. 8*D*; Extended Data Fig. 8-3). Rmdn3 was shown to be expressed in the Purkinje cell soma and dendrites (Koch et al., 2009) and is implied in calcium handling and interactions between mitochondria and the endoplasmic reticulum (Fecher et al., 2019). In the RNA sequence data, both *RMDN3 and HOMER3* genes are also upregulated in *PKC*γ*-A24E* mice (Fig. 8*E*). Western blotting confirmed the abundant expression of Rmdn3 and Homer3 in the cerebellum, with a slight upregulation in *PKC*γ*-A24E* (Fig. 8*F*). IPA network analysis shows that Dlgap1-Homer3-Shank1/3-Syne1-Pclo signaling is more phosphorylated in *PKC*γ*-A24E* mice, suggesting that postsynaptic scaffolding proteins might also be targets of PKCγ phosphorylation.

10.1523/JNEUROSCI.1946-20.2021.f8-3 Figure 8-3 Summary of phosphoproteomics analysis (symbol, gene name, fold changes, *p* values and locations). (A) 174 protein phosphorylations are significaltly changed in Homo PKCγ-A24E mice. 105 of 174 protein phosphorylations are significaltly increased while 69 of 174 protein phosphorylations are significaltly decreased in Homo PKCγ-A24E mice. Download Figure 8-3, DOCX file.

## Discussion

In this study, we present a new knock-in mouse model related to SCA14 with a constitutive activation by a mutated pseudosubstrate domain, which no longer can bind to the kinase domain and keeps the PKCγ protein in the open active conformation. This leads to a dramatic increase of dephosphorylation and protein degradation and to a drastic reduction of protein expression in cerebellar Purkinje cells. Despite this reduction in protein levels, there is clear evidence for an increased PKC activity in Purkinje cells from *PKC*γ*-A24E* mice with the typical morphology of short thickened dendrites, a marked ataxia, and signs of Purkinje cell dysfunction. A similar mutation in a human patient was associated with overt SCA14. Our results show that the introduction of a new mutation leading to a constant activation of PKCγ results in an SCA-like phenotype in these mice, establishing PKC activation as one pathogenetic avenue leading to an SCA phenotype.

### Increased PKC activity despite reduced protein levels of PKCγ-A24E because of increased degradation

We introduced mutations in the pseudosubstrate domain for abolishing self-inhibitory effect result in a constantly activated PKCγ enzyme (Pears et al., 1990; Newton, 2018). This concept was confirmed by transfecting pseudosubstrate domain mutated PKCγ in Purkinje cells, which showed a severe reduction of their dendritic tree consistent with a constitutive activation of PKCγ. Together with the increased PKC activation, we found a reduced amount of mutated PKCγ-A24E protein. Treatment with cycloheximide and qPCR studies confirmed that this reduction was because of increased degradation as expected for the protein in the “open active” conformation (Newton, 2018). In this conformation, the kinase is subject to dephosphorylation by protein phosphatases and to ubiquitination and degradation via the proteasomal pathway (Hansra et al., 1999). Our *in vitro* studies thus agree with the known regulation of PKC activation and degradation and show that the increased degradation of the open active protein in transfected cells did result in reduced protein levels. These findings are similar to those reported for the original A25E construct in PKCα (Pears et al., 1990).

### Dramatic reduction of PKCγ-A24E protein in the PKCγ-A24E mouse model is compatible with increased PKC activity

While the effect of pseudosubstrate mutations has been studied extensively with diverse PKC variants in cell culture assays, the *PKC*γ*-A24E* mouse is to our knowledge the first mouse model of such a pseudosubstrate mutation. We were surprised to find an almost complete absence of PKCγ-A24E protein in the cerebellum, but qPCR confirmed that mRNA levels were similar in all genotypes, suggesting that the reduction was because of increased degradation (Fig. 3). Despite this dramatic reduction of PKCγ-A24E protein, there was clear evidence for increased PKC activity. An antibody against an epitope recognizing PKC-mediated phosphorylation showed increased phosphorylation of target proteins both in Het and Homo *PKC*γ*-A24E* mice, and phosphorylation of the known target protein P890-NMDAR was also increased. The mice demonstrate that only a very small amount of active PKCγ is required for inducing increased PKC activity in a cell.

### Increased constitutive PKC activity interferes with Purkinje cell development and function

The increased constitutive activity of PKCγ-A24E has negative effects on Purkinje cells both in cell culture and *in vivo*. This is particularly evident in organotypic slice cultures and dissociated cerebellar cultures. In both cases, Purkinje cells only develop a small abnormal dendritic tree with thickened dendrites. In slice cultures, we could show that this effect is because of increased PKC activity because it can be rescued by the application of PKC inhibitor like Gö6983. When analyzed by Golgi staining, a mild reduction of the Purkinje cell dendritic tree size becomes evident. A similar difference has already been found in the PKCγ-S361G transgenic mouse (Ji et al., 2014; Trzesniewski et al., 2019). Importantly, also CF innervation is reduced, particularly in the distal parts of the Purkinje cell dendrites. This finding goes together with the known importance of Purkinje cell PKCγ activity for CF innervation (Chen et al., 1995) and corresponds to similar findings in the *PKC*γ*-S361G* transgenic mouse (Trzesniewski et al., 2019). The *PKC*γ*-A24E* mice have a marked ataxia, which is most evident with testing on the walking beam which some Homo *PKC*γ*-A24E* can barely cross; and also Het *PKC*γ*-A24E* mice have a clearly increased number of slips at both ages tested. The presence of the ataxia reflects a dysfunction of the neuronal cerebellar circuits controlling precision of movements and integration of vestibular information.

### The *PKC*γ*-A24E* mouse is a novel mouse model related to SCA14

The *PKC*γ*-A24E* mouse shows an ataxic phenotype but no extensive loss of Purkinje cells (data not shown). A similar situation applies to the *PKC*γ*-S361G* mouse (Ji et al., 2014). The presence of the ataxia in the absence of major Purkinje cell loss points to an important aspect of SCAs. While it is generally assumed that Purkinje cell loss is the major cause of the patients' problems, the evidence for this assumption is weak. Indeed, in mice a loss of 90% of Purkinje cells is required for the manifestation of overt motor behavioral deficits (Martin et al., 2003). In a recent study of SCA14 families, most patients had only mild to moderate atrophy of the cerebellum, making it doubtful that their ataxia can be explained exclusively by Purkinje cell loss. In the patient carrying the A24T mutation corresponding to the *PKC*γ*-A24E* mouse, only a mild cerebellar atrophy was found. In an SCA1 mouse model, the development of ataxia and Purkinje cell loss could be dissociated (Duvick et al., 2010), suggesting that Purkinje cell dysfunction is a crucial aspect for the development of the ataxic phenotype together with Purkinje cell loss. The increased PKC activity, the impairment of dendritic development, and the behavioral deficits make the Het *PKC*γ*-A24E* mice a valid mouse model related to SCA14.

### Changes in gene expression and phosphorylation in *PKC*γ*-A24E* mice

The mRNA profiling surprisingly yielded more results from the Het mice compared with the Homo mice. Many molecules in the oxidative phosphorylation pathway and mitochondrial molecules were upregulated in Het *PKC*γ*-A24E* mice (Fig. 8*A*,*B*; Extended Data Fig. 8-1*A*). Mitochondria trafficking into dendrites is essential for Purkinje cell dendritic outgrowth, and proper oxidative phosphorylation for energy production in mitochondria is very important for neuron activity. Indeed, mitochondrial dysfunction has been found in several neurodegenerative diseases, such as Alzheimer's disease (Friedland-Leuner et al., 2014), Parkinson's disease, Huntington's disease, and SCA1 (Stucki et al., 2016). In addition, the Rictor signaling pathway is strongly affected (Extended Data Fig. 8-1*C*). This pathway is well known to be of outstanding importance for Purkinje cell development and function (Angliker et al., 2015). In the Homo *PKC*γ*-A24E* mice, we find dysregulated genes involved in outgrowth and pruning of neuronal processes, such as the Eph receptors and changes in glutamate receptors and calcium homeostasis. This fits well with the idea that, in the *PKC*γ*-A24E* mice, the constitutive activation of PKCγ mimics a state of very strong synaptic activation, making it crucial for the Purkinje cells to handle the calcium release associated with this activation and limit the “natural” receptor activation through glutamate. With these compensatory mechanisms, the Purkinje cell would be stable and can survive, but it would be functionally compromised, resulting in the ataxic phenotype. This concept is also supported by the outcome of the phosphoproteomics analysis. Some of the most strongly phosphorylated proteins (e.g., Homer3 and Rmdn3) are involved in the control of receptor signaling and calcium handling; another one (Dpysl3) might control process outgrowth. Further studies will be required to further elucidate the exact role of these dysregulated proteins.

### PKCγ signaling and SCAs

In this manuscript, we show that the constitutive activation of PKCγ in the *PKC*γ*-A24E* mouse model is sufficient to induce a pathology related to SCA14, and this finding is supported by the recent identification of a human patient with a mutation at the same position (Chelban et al., 2018). However, other mutations causing SCA14 in humans affect the regulation of PKCγ differently or even are kinase dead (Shirafuji et al., 2019); and of course, most SCAs are caused by mutations in different genes. Nevertheless, slowly a picture is emerging that many of these mutations affect the mGluR1-PKCγ-inositol-1,4,5-trisphosphate receptor Type 1-calcium release pathway and can cause disease regardless of stimulation or inhibition of this pathway (Shimobayashi and Kapfhammer, 2018), meaning that it is not so important in which direction the activity if this pathway is pushed, but rather that the dynamic regulation of the activity of this pathway is disturbed. Although their overall activity pattern may look rather normal at first glance, Purkinje cells in these cases would be dysfunctional and the ataxic phenotype will become the more evident the more challenging the task is and can be present without noticeable Purkinje cell loss. Of course, in other cases, the loss of Purkinje cells rather than their dysfunction may be the key to SCA pathology. In the cases in which Purkinje cell dysfunction rather than death is at the base of the deficits, a pharmacological correction of the regulation of the affected pathway may be a valid therapeutic option.

## References

[B1] Adachi N, Kobayashi T, Takahashi H, Kawasaki T, Shirai Y, Ueyama T, Matsuda T, Seki T, Sakai N, Saito N (2008) Enzymological analysis of mutant protein kinase Cgamma causing spinocerebellar ataxia type 14 and dysfunction in Ca^2+^ homeostasis. J Biol Chem 283:19854–19863. 10.1074/jbc.M801492200 18499672

[B2] Angliker N, Burri M, Zaichuk M, Fritschy JM, Rüegg MA (2015) mTORC1 and mTORC2 have largely distinct functions in Purkinje cells. Eur J Neurosci 42:2595–2612. 10.1111/ejn.13051 26296489

[B3] Baffi TR, Van AN, Zhao W, Mills GB, Newton AC (2019) Protein kinase C quality control by phosphatase PHLPP1 unveils loss-of-function mechanism in cancer. Mol Cell 74:378–392.e375. 10.1016/j.molcel.2019.02.018 30904392 PMC6504549

[B4] Cesa R, Premoselli F, Renna A, Ethell IM, Pasquale EB, Strata P (2011) Eph receptors are involved in the activity-dependent synaptic wiring in the mouse cerebellar cortex. PLoS One 6:e19160. 10.1371/journal.pone.0019160 21559471 PMC3084771

[B5] Chelban V, Wiethoff S, Fabian-Jessing BK, Haridy NA, Khan A, Efthymiou S, Becker EB, O'Connor E, Hersheson J, Newland K, Hojland AT, Gregersen PA, Lindquist SG, Petersen MB, Nielsen JE, Nielsen M, Wood NW, Giunti P, Houlden H (2018) Genotype-phenotype correlations, dystonia and disease progression in spinocerebellar ataxia type 14. Mov Disord 33:1119–1129. 10.1002/mds.27334 29603387 PMC6175136

[B6] Chen C, Kano M, Abeliovich A, Chen L, Bao S, Kim JJ, Hashimoto K, Thompson RF, Tonegawa S (1995) Impaired motor coordination correlates with persistent multiple climbing fiber innervation in PKC gamma mutant mice. Cell 83:1233–1242. 10.1016/0092-8674(95)90148-5 8548809

[B7] Chen DH, Cimino PJ, Ranum LP, Zoghbi HY, Yabe I, Schut L, Margolis RL, Lipe HP, Feleke A, Matsushita M, Wolff J, Morgan C, Lau D, Fernandez M, Sasaki H, Raskind WH, Bird TD (2005) The clinical and genetic spectrum of spinocerebellar ataxia 14. Neurology 64:1258–1260. 10.1212/01.WNL.0000156801.64549.6B 15824357

[B8] Chen DH, Raskind WH, Bird TD (2012) Spinocerebellar ataxia type 14. Handb Clin Neurol 103:555–559. 10.1016/B978-0-444-51892-7.00036-X 21827914

[B9] Chopra R, Wasserman AH, Pulst SM, De Zeeuw CI, Shakkottai VG (2018) Protein kinase C activity is a protective modifier of Purkinje neuron degeneration in cerebellar ataxia. Hum Mol Genet 27:1396–1410. 10.1093/hmg/ddy050 29432535 PMC6251693

[B10] De Zeeuw CI, Hansel C, Bian F, Koekkoek SK, van Alphen AM, Linden DJ, Oberdick J (1998) Expression of a protein kinase C inhibitor in Purkinje cells blocks cerebellar LTD and adaptation of the vestibulo-ocular reflex. Neuron 20:495–508. 10.1016/S0896-6273(00)80990-3 9539124

[B11] Duvick L, Barnes J, Ebner B, Agrawal S, Andresen M, Lim J, Giesler GJ, Zoghbi HY, Orr HT (2010) SCA1-like disease in mice expressing wild-type ataxin-1 with a serine to aspartic acid replacement at residue 776. Neuron 67:929–935. 10.1016/j.neuron.2010.08.022 20869591 PMC2946945

[B12] Fecher C, Trovo L, Muller SA, Snaidero N, Wettmarshausen J, Heink S, Ortiz O, Wagner I, Kuhn R, Hartmann J, Karl RM, Konnerth A, Korn T, Wurst W, Merkler D, Lichtenthaler SF, Perocchi F, Misgeld T (2019) Cell-type-specific profiling of brain mitochondria reveals functional and molecular diversity. Nat Neurosci 22:1731–1742. 10.1038/s41593-019-0479-z 31501572

[B13] Friedland-Leuner K, Stockburger C, Denzer I, Eckert GP, Muller WE (2014) Mitochondrial dysfunction: cause and consequence of Alzheimer's disease. Prog Mol Biol Transl Sci 127:183–210. 25149218 10.1016/B978-0-12-394625-6.00007-6

[B14] Gugger OS, Hartmann J, Birnbaumer L, Kapfhammer JP (2012) P/Q-type and T-type calcium channels, but not type 3 transient receptor potential cation channels, are involved in inhibition of dendritic growth after chronic metabotropic glutamate receptor type 1 and protein kinase C activation in cerebellar Purkinje cells. Eur J Neurosci 35:20–33. 10.1111/j.1460-9568.2011.07942.x 22188405

[B15] Hansra G, Garcia-Paramio P, Prevostel C, Whelan RD, Bornancin F, Parker PJ (1999) Multisite dephosphorylation and desensitization of conventional protein kinase C isotypes. Biochem J 342:337–344. 10.1042/0264-6021:3420337 10455020 PMC1220470

[B16] Heintz TG, Eva R, Fawcett JW (2016) Regional regulation of Purkinje cell dendritic spines by integrins and Eph/ephrins. PLoS One 11:e0158558. 10.1371/journal.pone.0158558 27518800 PMC4982633

[B17] Hirai H (2018) Protein kinase C in the cerebellum: its significance and remaining conundrums. Cerebellum 17:23–27. 10.1007/s12311-017-0898-x 29134360

[B18] Ichikawa R, Hashimoto K, Miyazaki T, Uchigashima M, Yamasaki M, Aiba A, Kano M, Watanabe M (2016) Territories of heterologous inputs onto Purkinje cell dendrites are segregated by mGluR1-dependent parallel fiber synapse elimination. Proc Natl Acad Sci USA 113:2282–2287. 10.1073/pnas.1511513113 26858447 PMC4776453

[B19] Ji J, Hassler ML, Shimobayashi E, Paka N, Streit R, Kapfhammer JP (2014) Increased protein kinase C gamma activity induces Purkinje cell pathology in a mouse model of spinocerebellar ataxia 14. Neurobiol Dis 70:1–11. 10.1016/j.nbd.2014.06.002 24937631

[B20] Kano M, Hashimoto K, Chen C, Abeliovich A, Aiba A, Kurihara H, Watanabe M, Inoue Y, Tonegawa S (1995) Impaired synapse elimination during cerebellar development in PKC gamma mutant mice. Cell 83:1223–1231. 10.1016/0092-8674(95)90147-7 8548808

[B21] Kano M, Watanabe T, Uesaka N, Watanabe M (2018) Multiple phases of climbing fiber synapse elimination in the developing cerebellum. Cerebellum 17:722–734. 10.1007/s12311-018-0964-z 30009357

[B22] Koch P, Viard M, Stenzinger A, Brobeil A, Tag C, Steger K, Wimmer M (2009) Expression profile of PTPIP51 in mouse brain. J Comp Neurol 517:892–905. 10.1002/cne.22201 19844996

[B23] Martin LA, Goldowitz D, Mittleman G (2003) The cerebellum and spatial ability: dissection of motor and cognitive components with a mouse model system. Eur J Neurosci 18:2002–2010. 10.1046/j.1460-9568.2003.02921.x 14622233

[B24] Menke DB (2013) Engineering subtle targeted mutations into the mouse genome. Genesis 51:605–618. 10.1002/dvg.22422 23913666

[B25] Metzger F, Kapfhammer JP (2000) Protein kinase C activity modulates dendritic differentiation of rat Purkinje cells in cerebellar slice cultures. Eur J Neurosci 12:1993–2005. 10.1046/j.1460-9568.2000.00086.x 10886339

[B26] Newton AC (2018) Protein kinase C: perfectly balanced. Crit Rev Biochem Mol Biol 53:208–230. 10.1080/10409238.2018.1442408 29513138 PMC5901981

[B27] Pears CJ, Kour G, House C, Kemp BE, Parker PJ (1990) Mutagenesis of the pseudosubstrate site of protein kinase C leads to activation. Eur J Biochem 194:89–94. 10.1111/j.1432-1033.1990.tb19431.x 2253627

[B28] Saito N, Shirai Y (2002) Protein kinase C gamma (PKC gamma): function of neuron specific isotype. J Biochem 132:683–687. 10.1093/oxfordjournals.jbchem.a003274 12417016

[B29] Sánchez-Pérez AM, Felipo V (2005) Serines 890 and 896 of the NMDA receptor subunit NR1 are differentially phosphorylated by protein kinase C isoforms. Neurochem Int 47:84–91. 10.1016/j.neuint.2005.04.011 15936117

[B30] Shimobayashi E, Kapfhammer JP (2018) Calcium signaling, PKC gamma, IP3R1 and CAR8 link spinocerebellar ataxias and Purkinje cell dendritic development. Curr Neuropharmacol 16:151–159. 10.2174/1570159X15666170529104000 28554312 PMC5883377

[B31] Shimobayashi E, Wagner W, Kapfhammer JP (2016) Carbonic anhydrase 8 expression in Purkinje cells is controlled by PKCgamma activity and regulates Purkinje cell dendritic growth. Mol Neurobiol 53:5149–5160. 10.1007/s12035-015-9444-3 26399641

[B32] Shirafuji T, Shimazaki H, Miyagi T, Ueyama T, Adachi N, Tanaka S, Hide I, Saito N, Sakai N (2019) Spinocerebellar ataxia type 14 caused by a nonsense mutation in the PRKCG gene. Mol Cell Neurosci 98:46–53. 10.1016/j.mcn.2019.05.005 31158466

[B33] Shuvaev AN, Horiuchi H, Seki T, Goenawan H, Irie T, Iizuka A, Sakai N, Hirai H (2011) Mutant PKCγ in spinocerebellar ataxia type 14 disrupts synapse elimination and long-term depression in Purkinje cells *in vivo*. J Neurosci 31:14324–14334. 10.1523/JNEUROSCI.5530-10.2011 21976518 PMC6623654

[B34] Stucki DM, Ruegsegger C, Steiner S, Radecke J, Murphy MP, Zuber B, Saxena S (2016) Mitochondrial impairments contribute to Spinocerebellar ataxia type 1 progression and can be ameliorated by the mitochondria-targeted antioxidant MitoQ. Free Radic Biol Med 97:427–440. 10.1016/j.freeradbiomed.2016.07.005 27394174

[B35] Trzesniewski J, Altmann S, Jager L, Kapfhammer JP (2019) Reduced Purkinje cell size is compatible with near normal morphology and function of the cerebellar cortex in a mouse model of spinocerebellar ataxia. Exp Neurol 311:205–212. 10.1016/j.expneurol.2018.10.004 30312605

[B36] Verbeek DS, Knight MA, Harmison GG, Fischbeck KH, Howell BW (2005) Protein kinase C gamma mutations in spinocerebellar ataxia 14 increase kinase activity and alter membrane targeting. Brain 128:436–442. 10.1093/brain/awh378 15618281

[B37] Verbeek DS, Goedhart J, Bruinsma L, Sinke RJ, Reits EA (2008) PKC gamma mutations in spinocerebellar ataxia type 14 affect C1 domain accessibility and kinase activity leading to aberrant MAPK signaling. J Cell Sci 121:2339–2349. 10.1242/jcs.027698 18577575

[B38] Wang Y, Wang Y, Zhang H, Gao Y, Huang C, Zhou A, Zhou Y, Li Y (2016) Sequential posttranslational modifications regulate PKC degradation. Mol Biol Cell 27:410–420. 10.1091/mbc.E15-09-0624 26564794 PMC4713141

[B39] Wong MM, Hoekstra SD, Vowles J, Watson LM, Fuller G, Nemeth AH, Cowley SA, Ansorge O, Talbot K, Becker EB (2018) Neurodegeneration in SCA14 is associated with increased PKCgamma kinase activity, mislocalization and aggregation. Acta Neuropathol Commun 6:99. 10.1186/s40478-018-0600-7 30249303 PMC6151931

[B40] Yang JS, Wei HX, Chen PP, Wu G (2018) Roles of Eph/ephrin bidirectional signaling in central nervous system injury and recovery. Exp Ther Med 15:2219–2227. 10.3892/etm.2018.5702 29456630 PMC5795627

