# Supplementary material for: A New Mouse Model Related to SCA14 Carrying a Pseudosubstrate Domain Mutation in PKCγ Shows Perturbed Purkinje Cell Maturation and Ataxic Motor Behavior
Source: J Neurosci. 2021 Mar 3;41(9):2053–68. doi: 10.1523/JNEUROSCI.1946-20.2021 (PMC7939089; doi:10.1523/JNEUROSCI.1946-20.2021)
Supplement: Figure 8-1 — Summary of the significantly changed genes in Het PKCγ-A24E mice (symbol, gene name, fold changes, p values and locations). (A) Many mitochondrial function related genes are upregulated in Het PKCγ-A24E mice. (B) Summary of the significantly changed genes related to glutamate receptor signaling pathway in Het PKCγ-A24E mice. (C) Summary of the significantly changed genes related to RICTOR signaling pathway in Het PKCγ-A24E mice in RNA sequeincing. Download Figure 8-1, DOCX file. [file ns-JN-RM-1946-20-s04.docx]

**Extended data Figure 8-1. Summary of the significantly changed genes in Het PKCγ-A24E mice**

(A) Summary of mitochondrial function pathways significantly changed in Het PKCγ-A24E mice

| **Symbol** | **Entrez Gene Name** | **Expr Log Ratio** | **Expr p-value** |
| --- | --- | --- | --- |
| COX4I2 | cytochrome c oxidase subunit 4I2 | 1.064 | 0.00011 |
| COX7A1 | cytochrome c oxidase subunit 7A1 | 0.613 | 0.000324 |
| UQCR11 | ubiquinol-cytochrome c reductase, complex III subunit XI | 0.55 | 0.000113 |
| ATP5F1D | ATP synthase F1 subunit delta | 0.549 | 0.000158 |
| UQCRQ | ubiquinol-cytochrome c reductase complex III subunit VII | 0.519 | 0.000122 |
| Atp5e | ATP synthase, H+ transporting, mitochondrial F1 complex, epsilon | 0.507 | 0.000543 |
| NDUFA13 | NADH:ubiquinone oxidoreductase subunit A13 | 0.504 | 0.000308 |
| COX6A1 | cytochrome c oxidase subunit 6A1 | 0.504 | 0.000442 |
| COX4I1 | cytochrome c oxidase subunit 4I1 | 0.491 | 0.000937 |
| COX5A | cytochrome c oxidase subunit 5A | 0.491 | 0.000362 |
| COX8A | cytochrome c oxidase subunit 8A | 0.485 | 0.000137 |
| NDUFA1 | NADH:ubiquinone oxidoreductase subunit A1 | 0.484 | 0.000282 |
| NDUFB10 | NADH:ubiquinone oxidoreductase subunit B10 | 0.47 | 0.00107 |
| NDUFA2 | NADH:ubiquinone oxidoreductase subunit A2 | 0.464 | 0.000961 |
| UQCR10 | ubiquinol-cytochrome c reductase, complex III subunit X | 0.462 | 0.000931 |
| NDUFS7 | NADH:ubiquinone oxidoreductase core subunit S7 | 0.459 | 0.000224 |
| PRDX5 | peroxiredoxin 5 | 0.459 | 0.000236 |
| NDUFA5 | NADH:ubiquinone oxidoreductase subunit A5 | 0.457 | 0.000344 |
| HSD17B10 | hydroxysteroid 17-beta dehydrogenase 10 | 0.456 | 0.00107 |
| NDUFB9 | NADH:ubiquinone oxidoreductase subunit B9 | 0.456 | 0.000428 |
| NDUFA3 | NADH:ubiquinone oxidoreductase subunit A3 | 0.448 | 0.00147 |
| NDUFS8 | NADH:ubiquinone oxidoreductase core subunit S8 | 0.442 | 0.000791 |
| COX6B1 | cytochrome c oxidase subunit 6B1 | 0.442 | 0.000498 |
| NDUFB11 | NADH:ubiquinone oxidoreductase subunit B11 | 0.433 | 0.000307 |
| PARK7 | Parkinsonism associated deglycase | 0.431 | 0.000207 |
| NDUFB8 | NADH:ubiquinone oxidoreductase subunit B8 | 0.43 | 0.000606 |
| NDUFB7 | NADH:ubiquinone oxidoreductase subunit B7 | 0.421 | 0.0013 |
| NDUFA8 | NADH:ubiquinone oxidoreductase subunit A8 | 0.414 | 0.00249 |
| ATP5MF | ATP synthase membrane subunit f | 0.412 | 0.000671 |
| ATP5PO | ATP synthase peripheral stalk subunit OSCP | 0.412 | 0.00149 |
| ATP5MC3 | ATP synthase membrane subunit c locus 3 | 0.411 | 0.000389 |
| CYC1 | cytochrome c1 | 0.404 | 0.000529 |
| NDUFB4 | NADH:ubiquinone oxidoreductase subunit B4 | 0.4 | 0.00381 |
| GPX4 | glutathione peroxidase 4 | 0.396 | 0.000618 |
| NDUFB3 | NADH:ubiquinone oxidoreductase subunit B3 | 0.393 | 0.000639 |
| NDUFA7 | NADH:ubiquinone oxidoreductase subunit A7 | 0.392 | 0.000788 |
| TXNRD2 | thioredoxin reductase 2 | 0.391 | 0.00122 |
| Cox6c | cytochrome c oxidase subunit 6C | 0.389 | 0.00187 |
| FIS1 | fission, mitochondrial 1 | 0.387 | 0.000713 |
| ATP5PD | ATP synthase peripheral stalk subunit d | 0.385 | 0.00176 |
| UQCRC1 | ubiquinol-cytochrome c reductase core protein 1 | 0.38 | 0.0021 |
| NDUFA12 | NADH:ubiquinone oxidoreductase subunit A12 | 0.375 | 0.000531 |
| NDUFV1 | NADH:ubiquinone oxidoreductase core subunit V1 | 0.356 | 0.00196 |
| COX7A2 | cytochrome c oxidase subunit 7A2 | 0.353 | 0.00231 |
| COX7B | cytochrome c oxidase subunit 7B | 0.352 | 0.00277 |
| UQCRB | ubiquinol-cytochrome c reductase binding protein | 0.35 | 0.0021 |
| NDUFA4 | NDUFA4 mitochondrial complex associated | 0.347 | 0.00316 |
| ATP5MC1 | ATP synthase membrane subunit c locus 1 | 0.347 | 0.0026 |
| SDHC | succinate dehydrogenase complex subunit C | 0.338 | 0.0027 |
| SURF1 | SURF1 cytochrome c oxidase assembly factor | 0.33 | 0.0014 |
| SDHB | succinate dehydrogenase complex iron sulfur subunit B | 0.328 | 0.00259 |
| NDUFAB1 | NADH:ubiquinone oxidoreductase subunit AB1 | 0.328 | 0.00431 |
| UQCRFS1 | ubiquinol-cytochrome c reductase, Rieske iron-sulfur polypeptide 1 | 0.321 | 0.00149 |
| NDUFB5 | NADH:ubiquinone oxidoreductase subunit B5 | 0.317 | 0.00139 |
| ATP5PF | ATP synthase peripheral stalk subunit F6 | 0.317 | 0.00456 |
| NDUFA6 | NADH:ubiquinone oxidoreductase subunit A6 | 0.314 | 0.00276 |
| NDUFV3 | NADH:ubiquinone oxidoreductase subunit V3 | 0.314 | 0.00263 |
| ATP5F1B | ATP synthase F1 subunit beta | 0.313 | 0.00916 |
| NDUFS2 | NADH:ubiquinone oxidoreductase core subunit S2 | 0.298 | 0.00341 |
| NDUFA10 | NADH:ubiquinone oxidoreductase subunit A10 | 0.297 | 0.00245 |
| NDUFS4 | NADH:ubiquinone oxidoreductase subunit S4 | 0.294 | 0.00527 |
| ATP5F1C | ATP synthase F1 subunit gamma | 0.265 | 0.0092 |
| NDUFA9 | NADH:ubiquinone oxidoreductase subunit A9 | 0.26 | 0.00592 |
| SDHD | succinate dehydrogenase complex subunit D | 0.259 | 0.00669 |
| NDUFS3 | NADH:ubiquinone oxidoreductase core subunit S3 | 0.255 | 0.00143 |
| PSEN2 | presenilin 2 | 0.252 | 0.00489 |
| TXN2 | thioredoxin 2 | 0.241 | 0.0056 |
| ATP5PB | ATP synthase peripheral stalk-membrane subunit b | 0.234 | 0.0099 |
| HTRA2 | HtrA serine peptidase 2 | 0.231 | 0.00824 |
| PRDX3 | peroxiredoxin 3 | 0.23 | 0.00871 |
| RHOT2 | ras homolog family member T2 | 0.215 | 0.00535 |
| VDAC3 | voltage dependent anion channel 3 | 0.214 | 0.00793 |
| MAPK8 | mitogen-activated protein kinase 8 | -0.25 | 0.00985 |
| Aph1c | aph1 homolog C, gamma secretase subunit | -0.346 | 0.00161 |
| LRRK2 | leucine rich repeat kinase 2 | -0.435 | 0.00093 |

(B) Summary of glutamate receptor related genes significantly changed in Het PKCγ-A24E mice

| **Symbol** | **Entrez Gene Name** | **Expr Log Ratio** | **Expr p-value** | **Location** |
| --- | --- | --- | --- | --- |
| SLC1A6 | solute carrier family 1 member 6 | 0.562 | 0.000239 | Plasma Membrane |
| HOMER2 | homer scaffold protein 2 | -0.783 | 0.000448 | Plasma Membrane |
| CAMK4 | calciumcalmodulin dependent protein kinase IV | -0.784 | 0.000493 | Nucleus |
| GRIN2A | glutamate ionotropic receptor NMDA type subunit 2A | -0.639 | 0.000701 | Plasma Membrane |
| GRIA2 | glutamate ionotropic receptor AMPA type subunit 2 | -0.62 | 0.000693 | Plasma Membrane |
| GRIN2B | glutamate ionotropic receptor NMDA type subunit 2B | -0.737 | 0.000237 | Plasma Membrane |
| GRM5 | glutamate metabotropic receptor 5 | -0.906 | 0.0000939 | Plasma Membrane |

(C) Summary of the significantly changed genes related to RICTOR signaling pathway in Het PKCγ-A24E mice

| **Symbol** | **Entrez Gene Name** | **Expr Log Ratio** | **Expr p-value** | **Location** |
| --- | --- | --- | --- | --- |
| AR | androgen receptor | -0.4596 | 0.003262 | Nucleus |
| Atp5e | ATP synthase, H+ transporting, mitochondrial F1 complex, epsilon subunit | 0.507311 | 0.000543 | Cytoplasm |
| ATP5F1B | ATP synthase F1 subunit beta | 0.312575 | 0.009163 | Cytoplasm |
| ATP5F1C | ATP synthase F1 subunit gamma | 0.265217 | 0.009198 | Cytoplasm |
| ATP5F1D | ATP synthase F1 subunit delta | 0.548879 | 0.000158 | Cytoplasm |
| ATP5MC1 | ATP synthase membrane subunit c locus 1 | 0.347165 | 0.002603 | Cytoplasm |
| ATP5MC3 | ATP synthase membrane subunit c locus 3 | 0.411184 | 0.000389 | Cytoplasm |
| ATP5MF | ATP synthase membrane subunit f | 0.411556 | 0.000671 | Cytoplasm |
| ATP5PB | ATP synthase peripheral stalk-membrane subunit b | 0.233659 | 0.009898 | Cytoplasm |
| ATP5PD | ATP synthase peripheral stalk subunit d | 0.384705 | 0.001757 | Cytoplasm |
| ATP5PF | ATP synthase peripheral stalk subunit F6 | 0.317148 | 0.004557 | Cytoplasm |
| ATP5PO | ATP synthase peripheral stalk subunit OSCP | 0.412301 | 0.001488 | Cytoplasm |
| ATP6V0B | ATPase H+ transporting V0 subunit b | 0.339963 | 0.000812 | Cytoplasm |
| ATP6V0C | ATPase H+ transporting V0 subunit c | 0.364515 | 0.000707 | Cytoplasm |
| ATP6V0D1 | ATPase H+ transporting V0 subunit d1 | 0.290482 | 0.008027 | Cytoplasm |
| ATP6V0E2 | ATPase H+ transporting V0 subunit e2 | 0.256808 | 0.009663 | Cytoplasm |
| ATP6V1E1 | ATPase H+ transporting V1 subunit E1 | 0.26662 | 0.006813 | Cytoplasm |
| ATP6V1F | ATPase H+ transporting V1 subunit F | 0.417688 | 0.000665 | Cytoplasm |
| ATP6V1G1 | ATPase H+ transporting V1 subunit G1 | 0.327185 | 0.004504 | Cytoplasm |
| ATP6V1G2 | ATPase H+ transporting V1 subunit G2 | 0.292968 | 0.006674 | Cytoplasm |
| BAD | BCL2 associated agonist of cell death | 0.449088 | 0.000382 | Cytoplasm |
| BAX | BCL2 associated X, apoptosis regulator | 0.37429 | 0.000524 | Cytoplasm |
| BSG | basigin (Ok blood group) | 0.327296 | 0.005658 | Plasma Membrane |
| CFLAR | CASP8 and FADD like apoptosis regulator | -0.46339 | 0.001193 | Cytoplasm |
| COX4I1 | cytochrome c oxidase subunit 4I1 | 0.490884 | 0.000937 | Cytoplasm |
| COX4I2 | cytochrome c oxidase subunit 4I2 | 1.063742 | 0.00011 | Cytoplasm |
| COX5A | cytochrome c oxidase subunit 5A | 0.491025 | 0.000362 | Cytoplasm |
| Cox5b | cytochrome c oxidase subunit 5B | 0.492878 | 0.000191 | Cytoplasm |
| COX6A1 | cytochrome c oxidase subunit 6A1 | 0.504084 | 0.000442 | Cytoplasm |
| COX6B1 | cytochrome c oxidase subunit 6B1 | 0.442247 | 0.000498 | Cytoplasm |
| Cox6c | cytochrome c oxidase subunit 6C | 0.389225 | 0.001874 | Cytoplasm |
| COX7A1 | cytochrome c oxidase subunit 7A1 | 0.613164 | 0.000324 | Cytoplasm |
| COX7A2 | cytochrome c oxidase subunit 7A2 | 0.353384 | 0.00231 | Cytoplasm |
| COX7B | cytochrome c oxidase subunit 7B | 0.352053 | 0.002768 | Cytoplasm |
| Cox7c | cytochrome c oxidase subunit 7C | 0.351538 | 0.001612 | Cytoplasm |
| COX8A | cytochrome c oxidase subunit 8A | 0.484956 | 0.000137 | Cytoplasm |
| CYC1 | cytochrome c1 | 0.403544 | 0.000529 | Cytoplasm |
| FAU | FAU ubiquitin like and ribosomal protein S30 fusion | 0.480277 | 0.000928 | Cytoplasm |
| HIF1A | hypoxia inducible factor 1 subunit alpha | -0.36553 | 0.002281 | Nucleus |
| IFI16 | interferon gamma inducible protein 16 | -0.6703 | 0.000967 | Nucleus |
| MRPL13 | mitochondrial ribosomal protein L13 | 0.276296 | 0.009235 | Cytoplasm |
| NCAM2 | neural cell adhesion molecule 2 | -0.97932 | 0.000136 | Plasma Membrane |
| NDUFA1 | NADH:ubiquinone oxidoreductase subunit A1 | 0.483838 | 0.000282 | Cytoplasm |
| NDUFA10 | NADH:ubiquinone oxidoreductase subunit A10 | 0.29726 | 0.002453 | Cytoplasm |
| NDUFA2 | NADH:ubiquinone oxidoreductase subunit A2 | 0.46434 | 0.000961 | Cytoplasm |
| NDUFA3 | NADH:ubiquinone oxidoreductase subunit A3 | 0.44802 | 0.001466 | Cytoplasm |
| NDUFA4 | NDUFA4 mitochondrial complex associated | 0.347134 | 0.003155 | Cytoplasm |
| NDUFA5 | NADH:ubiquinone oxidoreductase subunit A5 | 0.457424 | 0.000344 | Cytoplasm |
| NDUFA6 | NADH:ubiquinone oxidoreductase subunit A6 | 0.313658 | 0.002757 | Cytoplasm |
| NDUFA7 | NADH:ubiquinone oxidoreductase subunit A7 | 0.392295 | 0.000788 | Cytoplasm |
| NDUFA8 | NADH:ubiquinone oxidoreductase subunit A8 | 0.414371 | 0.002492 | Cytoplasm |
| NDUFA9 | NADH:ubiquinone oxidoreductase subunit A9 | 0.260051 | 0.005921 | Cytoplasm |
| NDUFAB1 | NADH:ubiquinone oxidoreductase subunit AB1 | 0.328462 | 0.004313 | Cytoplasm |
| NDUFB10 | NADH:ubiquinone oxidoreductase subunit B10 | 0.469638 | 0.001072 | Cytoplasm |
| NDUFB3 | NADH:ubiquinone oxidoreductase subunit B3 | 0.392639 | 0.000639 | Cytoplasm |
| NDUFB4 | NADH:ubiquinone oxidoreductase subunit B4 | 0.399663 | 0.003809 | Cytoplasm |
| NDUFB5 | NADH:ubiquinone oxidoreductase subunit B5 | 0.316727 | 0.001385 | Cytoplasm |
| NDUFB7 | NADH:ubiquinone oxidoreductase subunit B7 | 0.420858 | 0.001295 | Cytoplasm |
| NDUFB8 | NADH:ubiquinone oxidoreductase subunit B8 | 0.429863 | 0.000606 | Cytoplasm |
| NDUFB9 | NADH:ubiquinone oxidoreductase subunit B9 | 0.455836 | 0.000428 | Cytoplasm |
| NDUFC1 | NADH:ubiquinone oxidoreductase subunit C1 | 0.391207 | 0.000308 | Cytoplasm |
| NDUFC2 | NADH:ubiquinone oxidoreductase subunit C2 | 0.346632 | 0.001454 | Cytoplasm |
| NDUFS2 | NADH:ubiquinone oxidoreductase core subunit S2 | 0.29831 | 0.003412 | Cytoplasm |
| NDUFS3 | NADH:ubiquinone oxidoreductase core subunit S3 | 0.254753 | 0.001434 | Cytoplasm |
| NDUFS4 | NADH:ubiquinone oxidoreductase subunit S4 | 0.293743 | 0.005267 | Cytoplasm |
| NDUFS7 | NADH:ubiquinone oxidoreductase core subunit S7 | 0.458755 | 0.000224 | Cytoplasm |
| NDUFS8 | NADH:ubiquinone oxidoreductase core subunit S8 | 0.441784 | 0.000791 | Cytoplasm |
| NDUFV1 | NADH:ubiquinone oxidoreductase core subunit V1 | 0.356003 | 0.001959 | Cytoplasm |
| NDUFV3 | NADH:ubiquinone oxidoreductase subunit V3 | 0.313969 | 0.002626 | Cytoplasm |
| POMP | proteasome maturation protein | 0.387223 | 0.001172 | Nucleus |
| PPA1 | inorganic pyrophosphatase 1 | 0.339515 | 0.000865 | Cytoplasm |
| PPA2 | inorganic pyrophosphatase 2 | 0.26493 | 0.002353 | Cytoplasm |
| PRKCA | protein kinase C alpha | -0.58409 | 0.003699 | Cytoplasm |
| PSMA5 | proteasome 20S subunit alpha 5 | 0.30401 | 0.006906 | Cytoplasm |
| PSMA7 | proteasome 20S subunit alpha 7 | 0.345483 | 0.000987 | Cytoplasm |
| PSMB1 | proteasome 20S subunit beta 1 | 0.336808 | 0.002588 | Cytoplasm |
| PSMB2 | proteasome 20S subunit beta 2 | 0.287396 | 0.006231 | Cytoplasm |
| PSMB4 | proteasome 20S subunit beta 4 | 0.419246 | 0.000536 | Cytoplasm |
| PSMB5 | proteasome 20S subunit beta 5 | 0.448582 | 0.000216 | Cytoplasm |
| PSMB6 | proteasome 20S subunit beta 6 | 0.43846 | 0.000722 | Nucleus |
| PSMB7 | proteasome 20S subunit beta 7 | 0.29293 | 0.004857 | Cytoplasm |
| PSMC1 | proteasome 26S subunit, ATPase 1 | 0.262069 | 0.007143 | Nucleus |
| PSMC3 | proteasome 26S subunit, ATPase 3 | 0.356657 | 0.001511 | Nucleus |
| PSMC4 | proteasome 26S subunit, ATPase 4 | 0.339083 | 0.001467 | Nucleus |
| PSMC5 | proteasome 26S subunit, ATPase 5 | 0.314625 | 0.003114 | Nucleus |
| PSMD13 | proteasome 26S subunit, non-ATPase 13 | 0.268749 | 0.003237 | Cytoplasm |
| PSMD14 | proteasome 26S subunit, non-ATPase 14 | 0.213914 | 0.006252 | Cytoplasm |
| PSMD4 | proteasome 26S subunit, non-ATPase 4 | 0.335742 | 0.001811 | Cytoplasm |
| PSMD7 | proteasome 26S subunit, non-ATPase 7 | 0.268824 | 0.002635 | Cytoplasm |
| PSMD8 | proteasome 26S subunit, non-ATPase 8 | 0.38581 | 0.000607 | Cytoplasm |
| PSME4 | proteasome activator subunit 4 | -0.27195 | 0.008294 | Cytoplasm |
| PTEN | phosphatase and tensin homolog | -0.47481 | 0.000445 | Cytoplasm |
| RICTOR | RPTOR independent companion of MTORC 2 | -0.47674 | 0.001985 | Cytoplasm |
| RPL10 | ribosomal protein L10 | 0.29575 | 0.009879 | Cytoplasm |
| RPL10A | ribosomal protein L10a | 0.389174 | 0.000298 | Nucleus |
| RPL11 | ribosomal protein L11 | 0.363124 | 0.004831 | Cytoplasm |
| RPL13A | ribosomal protein L13a | 0.367793 | 0.002594 | Cytoplasm |
| RPL14 | ribosomal protein L14 | 0.343911 | 0.002245 | Cytoplasm |
| RPL18 | ribosomal protein L18 | 0.406841 | 0.001101 | Cytoplasm |
| RPL21 | ribosomal protein L21 | 0.348439 | 0.008909 | Cytoplasm |
| RPL23 | ribosomal protein L23 | 0.306112 | 0.009626 | Cytoplasm |
| Rpl23a | ribosomal protein L23A | 0.369744 | 0.001822 | Nucleus |
| RPL26 | ribosomal protein L26 | 0.355381 | 0.003116 | Cytoplasm |
| Rpl29 | ribosomal protein L29 | 0.52096 | 0.005565 | Cytoplasm |
| Rpl34 | ribosomal protein L34 | 0.430303 | 0.005121 | Cytoplasm |
| RPL38 | ribosomal protein L38 | 0.479137 | 0.001147 | Cytoplasm |
| RPL4 | ribosomal protein L4 | 0.266775 | 0.007452 | Cytoplasm |
| RPL41 | ribosomal protein L41 | 0.512819 | 0.000686 | Cytoplasm |
| RPL6 | ribosomal protein L6 | 0.408076 | 0.003861 | Nucleus |
| RPL8 | ribosomal protein L8 | 0.409511 | 0.001346 | Cytoplasm |
| RPLP0 | ribosomal protein lateral stalk subunit P0 | 0.261515 | 0.008096 | Cytoplasm |
| Rplp1 | ribosomal protein, large, P1 | 0.41195 | 0.002359 | Nucleus |
| RPLP2 | ribosomal protein lateral stalk subunit P2 | 0.416521 | 0.000248 | Cytoplasm |
| RPS10 | ribosomal protein S10 | 0.381732 | 0.000912 | Cytoplasm |
| RPS13 | ribosomal protein S13 | 0.314335 | 0.004184 | Cytoplasm |
| RPS15 | ribosomal protein S15 | 0.422515 | 0.003209 | Cytoplasm |
| RPS19 | ribosomal protein S19 | 0.333197 | 0.00659 | Cytoplasm |
| RPS21 | ribosomal protein S21 | 0.331545 | 0.009873 | Cytoplasm |
| RPS26 | ribosomal protein S26 | 0.422493 | 0.000848 | Cytoplasm |
| RPS29 | ribosomal protein S29 | 0.367668 | 0.004992 | Cytoplasm |
| RPS5 | ribosomal protein S5 | 0.35798 | 0.001682 | Cytoplasm |
| RPS6 | ribosomal protein S6 | 0.287767 | 0.004796 | Cytoplasm |
| RPS8 | ribosomal protein S8 | 0.397825 | 0.001268 | Cytoplasm |
| RPS9 | ribosomal protein S9 | 0.36274 | 0.004895 | Cytoplasm |
| RSL24D1 | ribosomal L24 domain containing 1 | 0.281918 | 0.008804 | Nucleus |
| SDHB | succinate dehydrogenase complex iron sulfur subunit B | 0.327947 | 0.002586 | Cytoplasm |
| SDHC | succinate dehydrogenase complex subunit C | 0.337559 | 0.002697 | Cytoplasm |
| SDHD | succinate dehydrogenase complex subunit D | 0.258527 | 0.006695 | Cytoplasm |
| SEM1 | SEM1 26S proteasome complex subunit | 0.401838 | 0.00142 | Nucleus |
| Uba52 | ubiquitin A-52 residue ribosomal protein fusion product 1 | 0.473561 | 0.000638 | Cytoplasm |
| UQCR10 | ubiquinol-cytochrome c reductase, complex III subunit X | 0.462334 | 0.000931 | Cytoplasm |
| UQCR11 | ubiquinol-cytochrome c reductase, complex III subunit XI | 0.550222 | 0.000113 | Cytoplasm |
| UQCRB | ubiquinol-cytochrome c reductase binding protein | 0.350191 | 0.002099 | Cytoplasm |
| UQCRC1 | ubiquinol-cytochrome c reductase core protein 1 | 0.379601 | 0.002103 | Cytoplasm |
| UQCRFS1 | ubiquinol-cytochrome c reductase, Rieske iron-sulfur polypeptide 1 | 0.320886 | 0.001491 | Cytoplasm |
| UQCRHL | ubiquinol-cytochrome c reductase hinge protein like | 0.398906 | 0.002343 | Cytoplasm |
| UQCRQ | ubiquinol-cytochrome c reductase complex III subunit VII | 0.519167 | 0.000122 | Cytoplasm |
| VCAM1 | vascular cell adhesion molecule 1 | -0.42365 | 0.00507 | Plasma Membrane |
