# Supplementary material for: A New Mouse Model Related to SCA14 Carrying a Pseudosubstrate Domain Mutation in PKCγ Shows Perturbed Purkinje Cell Maturation and Ataxic Motor Behavior
Source: J Neurosci. 2021 Mar 3;41(9):2053–68. doi: 10.1523/JNEUROSCI.1946-20.2021 (PMC7939089; doi:10.1523/JNEUROSCI.1946-20.2021)
Supplement: Figure 8-2 — Summary of the significantly changed genes in Homo PKCγ-A24E mice (symbol, gene name, fold changes, p values and locations). (A) Summary of the significantly changed genes related to Ehprin receptor signaling pathways in Homo PKCγ-A24E mice. (B) Summary of the significantly changed genes related to glutamate receptor signaling pathway in Homo PKCγ-A24E mice in RNA sequeincing. Download Figure 8-2, DOCX file. [file ns-JN-RM-1946-20-s05.docx]

**Extended data Figure 8-2. Summary of the significantly changed genes in Homo PKCγ-A24E mice**

(A) Summary of the significantly changed genes related to Ehprin receptor signaling pathways in Homo PKCγ-A24E mice

| **Symbol** | **Entrez Gene Name** | **Expr Log Ratio** | **Expr p-value** | **Location** |
| --- | --- | --- | --- | --- |
| ITGA2 | integrin subunit alpha 2 | 0.723 | 0.000793 | Plasma Membrane |
| EPHA2 | EPH receptor A2 | 0.594 | 0.0104 | Plasma Membrane |
| EPHA6 | EPH receptor A6 | 0.573 | 0.000755 | Plasma Membrane |
| EPHB2 | EPH receptor B2 | 0.558 | 0.000395 | Plasma Membrane |
| EPHA5 | EPH receptor A5 | 0.422 | 0.00959 | Plasma Membrane |
| NGEF | neuronal guanine nucleotide exchange factor | 0.398 | 0.00591 | Cytoplasm |
| KALRN | kalirin RhoGEF kinase | 0.356 | 0.00582 | Cytoplasm |
| GNG13 | G protein subunit gamma 13 | 0.344 | 0.0185 | Plasma Membrane |
| MRAS | muscle RAS oncogene homolog | 0.231 | 0.0165 | Plasma Membrane |
| SRC | SRC proto-oncogene, non-receptor tyrosine kinase | 0.225 | 0.0358 | Cytoplasm |
| GNAL | G protein subunit alpha L | 0.206 | 0.0329 | Cytoplasm |
| GNG2 | G protein subunit gamma 2 | -0.266 | 0.014 | Plasma Membrane |
| ROCK2 | Rho associated coiled-coil containing protein kinase 2 | -0.314 | 0.0236 | Cytoplasm |
| GRIN2A | glutamate ionotropic receptor NMDA type subunit 2A | -0.459 | 0.00638 | Plasma Membrane |
| RASD2 | RASD family member 2 | -0.464 | 0.0195 | Cytoplasm |
| PAK5 | p21 (RAC1) activated kinase 5 | -0.486 | 0.0105 | Nucleus |

(B) Summary of the significantly changed genes related to glutamate receptor signalling pathway in Homo PKCγ-A24E mice

| **Symbol** | **Entrez Gene Name** | **Expr Log Ratio** | **Expr p-value** | **Location** |
| --- | --- | --- | --- | --- |
| GRIN2A | glutamate ionotropic receptor NMDA type subunit 2A | -0.459 | 0.00638 | Plasma Membrane |
| GRID1 | glutamate ionotropic receptor delta type subunit 1 | 0.279 | 0.049 | Plasma Membrane |
| GRIK4 | glutamate ionotropic receptor kainate type subunit 4 | 0.316 | 0.00609 | Plasma Membrane |
| GRIA1 | glutamate ionotropic receptor AMPA type subunit 1 | 0.329 | 0.0356 | Plasma Membrane |
| NEFH | neurofilament heavy | 0.334 | 0.0145 | Cytoplasm |
| GRIK3 | glutamate ionotropic receptor kainate type subunit 3 | 0.355 | 0.0277 | Plasma Membrane |
| CACNA1S | calcium voltage-gated channel subunit alpha1 S | 0.704 | 0.01 | Plasma Membrane |
