# Supplementary material for: A New Mouse Model Related to SCA14 Carrying a Pseudosubstrate Domain Mutation in PKCγ Shows Perturbed Purkinje Cell Maturation and Ataxic Motor Behavior
Source: J Neurosci. 2021 Mar 3;41(9):2053–68. doi: 10.1523/JNEUROSCI.1946-20.2021 (PMC7939089; doi:10.1523/JNEUROSCI.1946-20.2021)
Supplement: Figure 8-3 — Summary of phosphoproteomics analysis (symbol, gene name, fold changes, p values and locations). (A) 174 protein phosphorylations are significaltly changed in Homo PKCγ-A24E mice. 105 of 174 protein phosphorylations are significaltly increased while 69 of 174 protein phosphorylations are significaltly decreased in Homo PKCγ-A24E mice. Download Figure 8-3, DOCX file. [file ns-JN-RM-1946-20-s06.docx]

**Extended data Figure 8-3. List of the proteins which show significantly increased or decreased phosphorylation in PKCγ-A24E mice**

| **Symbol** | **Entrez Gene Name** | **Expr Log Ratio** | **Expr p-value** | **Location** |
| --- | --- | --- | --- | --- |
| INO80C | INO80 complex subunit C | 5.234 | 0.0247 | Nucleus |
| CFL1 | cofilin 1 | 4.745 | 0.0134 | Nucleus |
| DENND1A | DENN domain containing 1A | 4.524 | 0.0013 | Plasma Membrane |
| EIF3J | eukaryotic translation initiation factor 3 subunit J | 3.668 | 0.00901 | Cytoplasm |
| MAGI2 | membrane associated guanylate kinase, WW and PDZ domain containing 2 | 3.646 | 0.0372 | Plasma Membrane |
| NDRG1 | N-myc downstream regulated 1 | 3.474 | 0.0078 | Nucleus |
| MGLL | monoglyceride lipase | 3.401 | 0.0223 | Plasma Membrane |
| RMDN3 | regulator of microtubule dynamics 3 | 3.287 | 0.000073 | Cytoplasm |
| DLGAP1 | DLG associated protein 1 | 2.684 | 0.0058 | Plasma Membrane |
| UNC13A | unc-13 homolog A | 2.63 | 0.048 | Plasma Membrane |
| HUWE1 | HECT, UBA and WWE domain containing E3 ubiquitin protein ligase 1 | 2.29 | 0.0123 | Nucleus |
| INPP1 | inositol polyphosphate-1-phosphatase | 2.218 | 0.0209 | Cytoplasm |
| HOMER3 | homer scaffold protein 3 | 2.201 | 0.000216 | Plasma Membrane |
| RAP1GAP2 | RAP1 GTPase activating protein 2 | 2.173 | 0.0471 | Cytoplasm |
| DPYSL3 | dihydropyrimidinase like 3 | 2.012 | 0.0395 | Cytoplasm |
| MPRIP | myosin phosphatase Rho interacting protein | 1.854 | 0.0321 | Cytoplasm |
| PLPPR4 | phospholipid phosphatase related 4 | 1.832 | 0.00804 | Plasma Membrane |
| TJP2 | tight junction protein 2 | 1.774 | 0.0371 | Plasma Membrane |
| KCTD12 | potassium channel tetramerization domain containing 12 | 1.753 | 0.017 | Plasma Membrane |
| SLMAP | sarcolemma associated protein | 1.741 | 0.0114 | Plasma Membrane |
| SPATA6 | spermatogenesis associated 6 | 1.74 | 0.0242 | Cytoplasm |
| CCNL1 | cyclin L1 | 1.592 | 0.0414 | Nucleus |
| ADD2 | adducin 2 | 1.561 | 0.0194 | Cytoplasm |
| PSD2 | pleckstrin and Sec7 domain containing 2 | 1.552 | 0.0348 | Plasma Membrane |
| MATR3 | matrin 3 | 1.497 | 0.0286 | Nucleus |
| STAC | SH3 and cysteine rich domain | 1.389 | 0.0489 | Cytoplasm |
| TECR | trans-2,3-enoyl-CoA reductase | 1.365 | 0.0461 | Plasma Membrane |
| CHMP7 | charged multivesicular body protein 7 | 1.345 | 0.00404 | Cytoplasm |
| JAKMIP3 | Janus kinase and microtubule interacting protein 3 | 1.344 | 0.0084 | Other |
| KDM7A | lysine demethylase 7A | 1.333 | 0.0243 | Nucleus |
| SHANK1 | SH3 and multiple ankyrin repeat domains 1 | 1.318 | 0.0122 | Cytoplasm |
| SYNE1 | spectrin repeat containing nuclear envelope protein 1 | 1.302 | 0.0406 | Nucleus |
| CEP85 | centrosomal protein 85 | 1.297 | 0.0387 | Nucleus |
| TBC1D1 | TBC1 domain family member 1 | 1.29 | 0.0436 | Nucleus |
| S1PR1 | sphingosine-1-phosphate receptor 1 | 1.274 | 0.00898 | Plasma Membrane |
| KLC2 | kinesin light chain 2 | 1.26 | 0.0488 | Cytoplasm |
| CADPS | calcium dependent secretion activator | 1.218 | 0.0495 | Plasma Membrane |
| GORASP2 | golgi reassembly stacking protein 2 | 1.198 | 0.0323 | Cytoplasm |
| HRH1 | histamine receptor H1 | 1.188 | 0.0104 | Plasma Membrane |
| SCCPDH | saccharopine dehydrogenase (putative) | 1.184 | 0.00718 | Cytoplasm |
| DBN1 | drebrin 1 | 1.156 | 0.0449 | Cytoplasm |
| SRGAP2 | SLIT-ROBO Rho GTPase activating protein 2 | 1.133 | 0.0444 | Cytoplasm |
| Nefm | neurofilament, medium polypeptide | 1.122 | 0.034 | Plasma Membrane |
| PCLO | piccolo presynaptic cytomatrix protein | 1.099 | 0.0429 | Cytoplasm |
| AP2A1 | adaptor related protein complex 2 subunit alpha 1 | 1.065 | 0.0176 | Cytoplasm |
| MAP3K10 | mitogen-activated protein kinase kinase kinase 10 | 1.021 | 0.00729 | Cytoplasm |
| CEP76 | centrosomal protein 76 | 1.005 | 0.012 | Cytoplasm |
| CACNB2 | calcium voltage-gated channel auxiliary subunit beta 2 | 1.005 | 0.0194 | Plasma Membrane |
| CPEB3 | cytoplasmic polyadenylation element binding protein 3 | 0.982 | 0.00574 | Cytoplasm |
| SLCO4A1 | solute carrier organic anion transporter family member 4A1 | 0.95 | 0.0476 | Plasma Membrane |
| LARP1 | La ribonucleoprotein domain family member 1 | 0.947 | 0.0461 | Cytoplasm |
| CASP3 | caspase 3 | 0.937 | 0.0318 | Cytoplasm |
| BICD2 | BICD cargo adaptor 2 | 0.924 | 0.00971 | Cytoplasm |
| SPHKAP | SPHK1 interactor, AKAP domain containing | 0.9 | 0.00671 | Cytoplasm |
| DAGLA | diacylglycerol lipase alpha | 0.896 | 0.0155 | Plasma Membrane |
| THRAP3 | thyroid hormone receptor associated protein 3 | 0.879 | 0.0175 | Nucleus |
| SASH1 | SAM and SH3 domain containing 1 | 0.875 | 0.00733 | Extracellular Space |
| PRKAB2 | protein kinase AMP-activated non-catalytic subunit beta 2 | 0.87 | 0.0465 | Cytoplasm |
| MTMR2 | myotubularin related protein 2 | 0.868 | 0.0261 | Cytoplasm |
| CDK18 | cyclin dependent kinase 18 | 0.862 | 0.0463 | Cytoplasm |
| HELB | DNA helicase B | 0.835 | 0.0132 | Nucleus |
| VIPR2 | vasoactive intestinal peptide receptor 2 | 0.826 | 0.0146 | Plasma Membrane |
| MAP7D1 | MAP7 domain containing 1 | 0.82 | 0.00664 | Cytoplasm |
| MAP2 | microtubule associated protein 2 | 0.8 | 0.0309 | Plasma Membrane |
| GRM1 | glutamate metabotropic receptor 1 | 0.775 | 0.0057 | Plasma Membrane |
| SH3PXD2B | SH3 and PX domains 2B | 0.766 | 0.0437 | Cytoplasm |
| MAPT | microtubule associated protein tau | 0.757 | 0.0421 | Plasma Membrane |
| ARVCF | ARVCF delta catenin family member | 0.742 | 0.0241 | Plasma Membrane |
| SQSTM1 | sequestosome 1 | 0.74 | 0.0347 | Cytoplasm |
| SHANK3 | SH3 and multiple ankyrin repeat domains 3 | 0.731 | 0.0416 | Plasma Membrane |
| Ank2 | ankyrin 2, brain | 0.717 | 0.0214 | Plasma Membrane |
| OSBP | oxysterol binding protein | 0.702 | 0.0231 | Cytoplasm |
| EDC4 | enhancer of mRNA decapping 4 | 0.685 | 0.0241 | Cytoplasm |
| STRN3 | striatin 3 | 0.681 | 0.0238 | Nucleus |
| NECAP2 | NECAP endocytosis associated 2 | 0.677 | 0.0343 | Cytoplasm |
| SPEG | striated muscle enriched protein kinase | 0.674 | 0.0359 | Nucleus |
| PPP2R5B | protein phosphatase 2 regulatory subunit B'beta | 0.67 | 0.0215 | Cytoplasm |
| USP20 | ubiquitin specific peptidase 20 | 0.668 | 0.0484 | Cytoplasm |
| CBL | Cbl proto-oncogene | 0.655 | 0.026 | Nucleus |
| TRIO | trio Rho guanine nucleotide exchange factor | 0.648 | 0.0421 | Cytoplasm |
| SERINC1 | serine incorporator 1 | 0.64 | 0.0087 | Plasma Membrane |
| ABCF1 | ATP binding cassette subfamily F member 1 | 0.636 | 0.0463 | Cytoplasm |
| EPB41L3 | erythrocyte membrane protein band 4.1 like 3 | 0.623 | 0.0384 | Plasma Membrane |
| ZSWIM8 | zinc finger SWIM-type containing 8 | 0.614 | 0.0298 | Extracellular Space |
| USP24 | ubiquitin specific peptidase 24 | 0.611 | 0.0217 | Nucleus |
| PTPRZ1 | protein tyrosine phosphatase receptor type Z1 | 0.611 | 0.0176 | Plasma Membrane |
| SLC25A46 | solute carrier family 25 member 46 | 0.609 | 0.0461 | Cytoplasm |
| SLC6A5 | solute carrier family 6 member 5 | 0.608 | 0.0172 | Plasma Membrane |
| JUN | Jun proto-oncogene, AP-1 transcription factor subunit | 0.6 | 0.0217 | Nucleus |
| TRIM32 | tripartite motif containing 32 | 0.592 | 0.0279 | Nucleus |
| NCAM1 | neural cell adhesion molecule 1 | 0.589 | 0.0324 | Plasma Membrane |
| AEBP2 | AE binding protein 2 | 0.563 | 0.0432 | Nucleus |
| LUC7L | LUC7 like | 0.552 | 0.049 | Nucleus |
| WASHC2A/WASHC2C | WASH complex subunit 2A | 0.547 | 0.0461 | Cytoplasm |
| MAPK8IP3 | mitogen-activated protein kinase 8 interacting protein 3 | 0.542 | 0.047 | Cytoplasm |
| ARHGAP20 | Rho GTPase activating protein 20 | 0.531 | 0.0475 | Cytoplasm |
| ATXN2L | ataxin 2 like | 0.528 | 0.0333 | Nucleus |
| EIF4G1 | eukaryotic translation initiation factor 4 gamma 1 | 0.519 | 0.0469 | Cytoplasm |
| TNKS1BP1 | tankyrase 1 binding protein 1 | 0.517 | 0.0225 | Nucleus |
| MLC1 | modulator of VRAC current 1 | 0.516 | 0.0323 | Plasma Membrane |
| TSC22D4 | TSC22 domain family member 4 | 0.493 | 0.0465 | Nucleus |
| GRIP1 | glutamate receptor interacting protein 1 | 0.49 | 0.0466 | Plasma Membrane |
| DACT3 | dishevelled binding antagonist of beta catenin 3 | 0.476 | 0.0442 | Cytoplasm |
| PPP1R7 | protein phosphatase 1 regulatory subunit 7 | 0.466 | 0.0403 | Nucleus |
| UNC79 | unc-79 homolog, NALCN channel complex subunit | 0.424 | 0.0435 | Other |
| PREX1 | phosphatidylinositol-3,4,5-trisphosphate dependent Rac exchange factor 1 | -0.487 | 0.0386 | Cytoplasm |
| NR3C1 | nuclear receptor subfamily 3 group C member 1 | -0.527 | 0.0411 | Nucleus |
| KRI1 | KRI1 homolog | -0.547 | 0.0318 | Nucleus |
| GJA1 | gap junction protein alpha 1 | -0.561 | 0.0295 | Plasma Membrane |
| DNAJC17 | DnaJ heat shock protein family (Hsp40) member C17 | -0.562 | 0.0374 | Other |
| ARHGAP44 | Rho GTPase activating protein 44 | -0.571 | 0.0304 | Cytoplasm |
| SRF | serum response factor | -0.579 | 0.0477 | Nucleus |
| ENO1 | enolase 1 | -0.582 | 0.0432 | Cytoplasm |
| ACACA | acetyl-CoA carboxylase alpha | -0.606 | 0.0397 | Cytoplasm |
| AARSD1 | alanyl-tRNA synthetase domain containing 1 | -0.627 | 0.0222 | Nucleus |
| WNK1 | WNK lysine deficient protein kinase 1 | -0.66 | 0.027 | Cytoplasm |
| HNRNPA2B1 | heterogeneous nuclear ribonucleoprotein A2/B1 | -0.692 | 0.0197 | Nucleus |
| FAM122A | family with sequence similarity 122A | -0.698 | 0.0499 | Extracellular Space |
| SLC9A3R1 | SLC9A3 regulator 1 | -0.716 | 0.0316 | Plasma Membrane |
| AJM1 | apical junction component 1 homolog | -0.723 | 0.0271 | Other |
| SHISA6 | shisa family member 6 | -0.771 | 0.0194 | Plasma Membrane |
| SLC4A7 | solute carrier family 4 member 7 | -0.773 | 0.0248 | Plasma Membrane |
| Srrm1 | serine/arginine repetitive matrix 1 | -0.774 | 0.00734 | Nucleus |
| ANO3 | anoctamin 3 | -0.799 | 0.0196 | Plasma Membrane |
| STXBP1 | syntaxin binding protein 1 | -0.814 | 0.0335 | Cytoplasm |
| RAB3A | RAB3A, member RAS oncogene family | -0.819 | 0.0307 | Cytoplasm |
| NDRG4 | NDRG family member 4 | -0.827 | 0.00713 | Plasma Membrane |
| POLR2A | RNA polymerase II subunit A | -0.843 | 0.0332 | Nucleus |
| FRMD4B | FERM domain containing 4B | -0.847 | 0.0396 | Cytoplasm |
| COBLL1 | cordon-bleu WH2 repeat protein like 1 | -0.866 | 0.032 | Extracellular Space |
| SENP7 | SUMO specific peptidase 7 | -0.872 | 0.0287 | Nucleus |
| SPECC1 | sperm antigen with calponin homology and coiled-coil domains 1 | -0.913 | 0.0294 | Nucleus |
| GJC3 | gap junction protein gamma 3 | -0.954 | 0.0178 | Extracellular Space |
| DDX21 | DExD-box helicase 21 | -0.961 | 0.0428 | Nucleus |
| NOP56 | NOP56 ribonucleoprotein | -0.991 | 0.0299 | Nucleus |
| ARHGAP1 | Rho GTPase activating protein 1 | -1.023 | 0.00894 | Cytoplasm |
| PRKG1 | protein kinase cGMP-dependent 1 | -1.026 | 0.00596 | Cytoplasm |
| GPM6A | glycoprotein M6A | -1.034 | 0.0323 | Plasma Membrane |
| SLC7A14 | solute carrier family 7 member 14 | -1.036 | 0.0496 | Cytoplasm |
| ACSS2 | acyl-CoA synthetase short chain family member 2 | -1.048 | 0.0447 | Cytoplasm |
| EHD2 | EH domain containing 2 | -1.062 | 0.0401 | Nucleus |
| LDB1 | LIM domain binding 1 | -1.085 | 0.0401 | Nucleus |
| PLIN1 | perilipin 1 | -1.115 | 0.0384 | Cytoplasm |
| ULK2 | unc-51 like autophagy activating kinase 2 | -1.141 | 0.0105 | Cytoplasm |
| ATG4B | autophagy related 4B cysteine peptidase | -1.199 | 0.0104 | Cytoplasm |
| SPARCL1 | SPARC like 1 | -1.296 | 0.00743 | Extracellular Space |
| APBA1 | amyloid beta precursor protein binding family A member 1 | -1.339 | 0.0269 | Cytoplasm |
| GABBR2 | gamma-aminobutyric acid type B receptor subunit 2 | -1.36 | 0.0476 | Plasma Membrane |
| AVEN | apoptosis and caspase activation inhibitor | -1.362 | 0.0128 | Nucleus |
| TRA2B | transformer 2 beta homolog | -1.505 | 0.013 | Nucleus |
| RTN4 | reticulon 4 | -1.535 | 0.00714 | Cytoplasm |
| SLC4A4 | solute carrier family 4 member 4 | -1.557 | 0.0306 | Plasma Membrane |
| KIF1A | kinesin family member 1A | -1.568 | 0.0489 | Cytoplasm |
| COIL | coilin | -1.574 | 0.0359 | Nucleus |
| Anp32a | acidic (leucine-rich) nuclear phosphoprotein 32 family, member A | -1.615 | 0.0334 | Nucleus |
| ZC3H18 | zinc finger CCCH-type containing 18 | -1.733 | 0.0228 | Nucleus |
| UNC13C | unc-13 homolog C | -1.784 | 0.0064 | Cytoplasm |
| DENND5A | DENN domain containing 5A | -1.835 | 0.00944 | Cytoplasm |
| CAMK2A | calcium/calmodulin dependent protein kinase II alpha | -1.902 | 0.0301 | Cytoplasm |
| ARHGEF33 | Rho guanine nucleotide exchange factor 33 | -1.99 | 0.0388 | Other |
| ITSN1 | intersectin 1 | -2.062 | 0.0472 | Cytoplasm |
| TACC2 | transforming acidic coiled-coil containing protein 2 | -2.304 | 0.0246 | Nucleus |
| PLP1 | proteolipid protein 1 | -2.864 | 0.0265 | Plasma Membrane |
| BSN | bassoon presynaptic cytomatrix protein | -3.657 | 0.0441 | Plasma Membrane |
| LARP7 | La ribonucleoprotein domain family member 7 | -3.773 | 0.0387 | Nucleus |
| MRVI1 | murine retrovirus integration site 1 homolog | -3.908 | 0.0282 | Cytoplasm |
| STRN | striatin | -4.001 | 0.00413 | Cytoplasm |
| Zrsr1 | zinc finger (CCCH type), RNA binding motif and serine/arginine rich 1 | -4.202 | 0.0462 | Cytoplasm |
| DMXL2 | Dmx like 2 | -4.452 | 0.0131 | Cytoplasm |
| NEFH | neurofilament heavy | -5.259 | 1.66E-07 | Cytoplasm |
| SERBP1 | SERPINE1 mRNA binding protein 1 | -5.302 | 0.0258 | Cytoplasm |
| CBX3 | chromobox 3 | -5.793 | 0.0404 | Nucleus |
| SAFB | scaffold attachment factor B | -9.224 | 0.0198 | Nucleus |
| PRKCG | protein kinase C gamma | -10.077 | 0.0317 | Cytoplasm |
